# The carbon costs of global wood harvests

**DOI:** 10.1038/s41586-023-06187-1

**Published:** 2023-07-05

**Authors:** Liqing Peng, Timothy D. Searchinger, Jessica Zionts, Richard Waite

**Affiliations:** 1 World Resources Institute, Washington, DC USA; 2 Princeton University, Princeton, NJ USA

**Keywords:** Environmental sciences, Climate-change mitigation

## Abstract

After agriculture, wood harvest is the human activity that has most reduced the storage of carbon in vegetation and soils^1,2^. Although felled wood releases carbon to the atmosphere in various steps, the fact that growing trees absorb carbon has led to different carbon-accounting approaches for wood use, producing widely varying estimates of carbon costs. Many approaches give the impression of low, zero or even negative greenhouse gas emissions from wood harvests because, in different ways, they offset carbon losses from new harvests with carbon sequestration from growth of broad forest areas^3,4^. Attributing this sequestration to new harvests is inappropriate because this other forest growth would occur regardless of new harvests and typically results from agricultural abandonment, recovery from previous harvests and climate change itself. Nevertheless some papers count gross emissions annually, which assigns no value to the capacity of newly harvested forests to regrow and approach the carbon stocks of unharvested forests. Here we present results of a new model that uses time discounting to estimate the present and future carbon costs of global wood harvests under different scenarios. We find that forest harvests between 2010 and 2050 will probably have annualized carbon costs of 3.5–4.2 Gt CO_2_e yr^−1^, which approach common estimates of annual emissions from land-use change due to agricultural expansion. Our study suggests an underappreciated option to address climate change by reducing these costs.

## Abstract (web summary)

A new model uses time discounting to estimate the present and future carbon costs of global wood harvests under different scenarios, suggesting an underappreciated option to address climate change.

## Main

The greenhouse gas (GHG) effects of forest harvests are accounted for in different contexts: lifecycle calculations of wood products, national reporting of GHGs by governments and scientific analyses assessing emissions from land-use change. Although details vary, the most frequent approaches share the common characteristic that carbon gains from regrowth of trees from previous human land management—and sometimes further growth of unharvested trees—cancel out carbon losses due to new harvests^3,4^. For example, life cycle analyses of wood products or wood-based bioenergy commonly treat uses of wood as ‘carbon neutral’ provided that the forests harvested are managed ‘sustainably’^3,5^. Carbon neutral means that they do not count the carbon that was present in vegetation (biogenic carbon) and emitted in various stages to the air as a result of harvest, such as from decomposing roots and slash and burning either for fuel, as waste or at end of use. Although sustainable is often not defined, a typical view endorsed in several lifecycle standards^3,5^ is that forest harvest is sustainable and carbon neutral provided  that harvests maintain carbon stocks by not exceeding the annual growth of the ‘forest’ (sometimes defined as a whole country). Some such studies also count the storage of even a small portion of this carbon in long-lived wood products as a carbon gain^3^. Some studies even attribute to wood harvest and use the average carbon stock in the forest stands providing the wood^6^. According to these last two variations, harvesting wood is not merely carbon neutral but adds to carbon storage and benefits the climate.

At the national level, countries report the effects of forestry using netting approaches that can create a similar impression of carbon neutrality. Because of the difficulty involved in separating the effects of human management from natural changes in forests, guidelines of the Intergovernmental Panel on Climate Change allow countries to report all changes in forest carbon stocks from ‘managed’ forests as emissions or removals of carbon from the atmosphere^4^. At 3 billion hectares, managed forests represent three-quarters of the world’s forests and in many countries nearly all^4^. These rules allow countries to ‘take credit’ for the regrowth of forests after agricultural abandonment or previous harvests (even harvests before international climate agreements that established 1990 as a base year)^7^. This approach also allows countries to claim credit from the large acceleration of growth in their forests due to CO_2_ fertilization effects, warmer weather and nitrogen deposition^8^. The harvest of wood according to this approach should reduce the nationally reported carbon sink but, because the effects of the harvest are not reported separately, these reports can give the impression that harvests in countries with net increases in forest carbon stocks have no emissions.

Unlike these national reports, scientific papers estimating emissions from land-use change attempt to factor out these effects of climate change on the forest carbon balance as the ‘residual land carbon sink’ but can still create a similar impression. That is because many papers report only the net effects of new wood harvests and regrowth from previous harvests and therefore do not identify the effects of new wood harvests alone^9–11^.

Each of these forms of accounting has strong regional implications. Most forests in temperate countries are recovering^4,8,12,13^ from vast harvesting or agricultural clearing in the past, aided by a reduced need to feed horses and other draught animals and outsourcing of farmland to the tropics^1,14–16^. By contrast, tropical countries overall have expanding farmland and increasing forest harvests^14,17^. Netting therefore can create the impression that wood harvests in temperate, developed countries have zero or even beneficial climate consequences whereas harvests in developing, tropical countries are costly^9,18,19^.

These forms of accounting do not accurately capture the effects of new forest harvests for the basic reason that the forest growth and regrowth used to offset the effects of new harvests would happen anyway^20^. As hundreds of scientists in letters and many scientific bodies have written, any growth or regrowth of forests that would occur anyway cannot logically alter the climate consequences of new harvests^21–24^.

On the other hand, some papers do report gross emissions from wood harvests^25–27^, often in papers about the tropics^28–31^. Although gross emissions matter too, they do not seem an adequate measure of the climate costs of harvests because they fail to account for potential regrowth after harvests. At some point, regrowing forests after harvest will probably start to recoup the lost carbon by growing faster than the same forests if left unharvested, even though they will rarely fully catch up.

These losses in the near- or medium-term—in addition to long-term losses—undermine the goals of the Paris Agreement and contradict the justifiable commitments many governments have made to achieve carbon neutrality by 2050 to avoid dangerous climate change^32^. Reflecting the importance of near-term emissions reductions, European and US governments have required that the climate effects of either direct or indirect land-use change due to bioenergy be judged over 20 or 30 years^33^. It makes sense to also place higher value on near-term emissions and mitigation when evaluating the effects of forest harvests.

## Accounting for time in estimation of GHG costs

Here we use time discounting to estimate the value of carbon losses due to past and probable future forest harvests from 2010 to 2050 under different supply-and-demand scenarios. We use a new global forest carbon model, the carbon harvest model (CHARM), which builds on a long-established approach^34^ of counting the effect of wood harvests on changes in atmospheric carbon over time as carbon shifts among different storage ‘pools’. Pools include live vegetation, roots, slash, different wood products and landfills. The effect on atmospheric carbon is the difference between carbon stored in all pools due to the harvest and the carbon that forests would store if left unharvested and continued to grow. In any given year, emissions to and removals from the air are the changes in this quantity from the previous year.

To value the cost of a tonne of emissions, equivalent in absolute value to a tonne of mitigation, our principal approach follows the method in ref. ^33^ and applies a discount rate of 4% to emissions and removals over time that result from each year’s wood harvest. For example, under this approach a tonne of carbon emitted in year 1 has a 4% higher absolute value than a tonne of carbon emitted or removed in year 2. In effect, this method translates the value of a flow of emissions and removals in future years resulting from a wood harvest into ‘harvest-year equivalent emissions’. If the discount rate is zero, the method estimates the absolute change in emissions in any given year after harvest.

For life cycle analyses of wood products, some previous stand analyses have accounted for time by calculating at a specific future date—such as 100 years—the cumulative radiative forcing resulting from a harvest, incorporating atmospheric decay rates of GHGs^3,12,35^. For biofuels, some papers have in turn applied a discount rate to these changes in radiative forcing as a proxy for climate damages^36,37^. Although informative, we believe these approaches disregard an important insight in the literature regarding the social cost of carbon (SCC), which estimates the changing real economic cost of emissions over time. Although similarly accounting for GHG decay rates, the SCC depends also on the costs of mitigation in the year of the emission. As a result, for example, assumptions that mitigation costs will decline over time due to new technology can lead to a declining SCC^38^. The intuitive reason is that if any product, such as a cell phone, has declining costs over time but is also needed now, one unit of that product (for example, the cell phone) is more valuable now than in the future. The value of a supply of goods over time—for example, mitigation—must also be discounted to reflect the time value of money. For both reasons the cost of a tonne of emissions, and correlatively the value of a tonne of mitigation, should vary with time.

Because different researchers estimate alternatively rising and declining changes in the SCC over time, our discount rate of 4% is consistent with a middle-ground estimate of a constant SCC and a 4% real rate of return on capital. A discounted cost of carbon can be thought of as the ‘rental cost’ of carbon. It also represents the interest one company would have to pay on funds used to buy offsets for its emissions until it paid back those emissions through subsequent mitigation. As discussed in ref. ^33^, this 4% rate provides a rigorous approach to time that reasonably matches real policies that value emissions from land-use change for biofuels. Our main results discount over 40 years, but we also discount over 100 years and vary discount rates from 0 to 6%.

## Growing wood demand

We start by projecting future wood consumption by country of four, broad categories of wood products: long-lived products (LLP), which are sawn wood and wood panels and other industrial roundwood; short-lived products (SLP), which are paper and paperboard products; very-short-lived products–wood fuel (VSLP–WFL), which is wood harvested deliberately for energy; and very-short-lived products–industrial (VSLP–IND), which is the waste from the manufacture of other wood products that is burned for energy. Our fixed-effects model estimates this consumption based on historical relationships among consumption of the major categories of wood products and population, country, gross domestic product (GDP) and time (as a proxy for technology change). The model, which assumes causality, obtains reasonable but imperfect fits and should be seen as one reasonable benchmark of future wood demand.

**Fig. 1:**
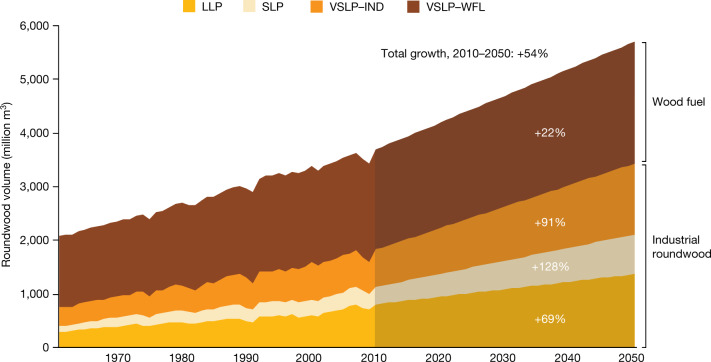
Historical and projected increases in global wood product production (million m^3^) between 1961 and 2050. This figure shows a projected 54% increase in global wood harvest from 2010 to 2050 based on a country fixed-effects model and illustrates separate growth in four separate categories of wood product. The model uses past relationships between consumption of each of those categories of wood and population, GDP per capita and time variables. The model applies the same relationship of wood consumption to each country’s estimated future population and per capita income growth, but starts with each country’s initial consumption of each category of wood product in recognition that countries have developed different reliance on wood in significant part because of different national endowments. Relationships are estimated after separation of countries into developed and developing countries, to avoid overestimation of future wood consumption in high-income countries. LLP includes sawn wood, wood panels and other industrial roundwood; SLP refers to paper and paperboard products; VSLP–IND refers to wastes of other wood product manufactured that are burned for energy; and VSLP–WFL refers to wood harvested to burn for energy. We consider VSLP-WFL projections the most uncertain because countries have shifted from traditional wood fuel to other energy sources at different income levels. Supplementary Information provides statistics on model fits.

On a global basis the model projects that wood harvests will increase by 54% between 2010 and 2050, from 3.7 billion m^3^ in 2010 to 5.7 billion m^3^ in 2050—a 69% increase in LLP, 128% increase in SLP, 22% increase in VSLP–WFL and 91% increase in VSLP–IND (Fig. 1). Our projected growth rates lie within the range of other studies, although they mostly provide shorter-term projections. We consider wood fuel projections the most uncertain because countries have shifted away from traditional bioenergy at different income levels.

**Fig. 2:**
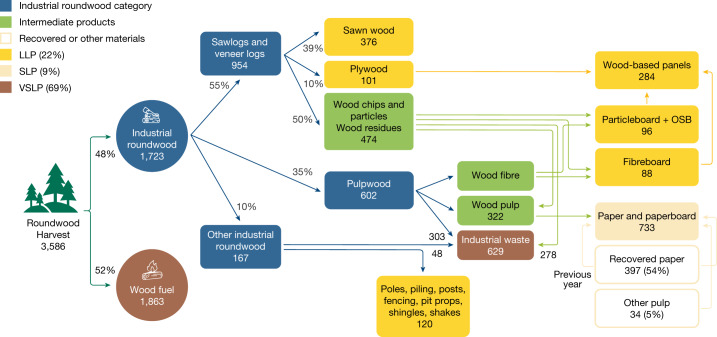
Flowchart of 2010 global roundwood (million m^3^), tracing the relationship between consumption of different wood products and wood harvests. We reconstruct the relationships between wood harvests and consumption of different wood products based on data from the UN Food and Agriculture Organization (FAO). The authors’ estimates from all sources are discussed in Methods and Supplementary Information. The flowchart illustrates the distribution of roundwood harvests into different wood products on a global basis in 2010. The numbers under wood products are volumes in cubic metres roundwood equivalents with the same density of raw wood harvests (0.48 tonnes dry matter m^–3^) for consistent interpretation. Once harvested, roundwood is either directly used as wood fuel (52%) or transported to the processing facilities as industrial roundwood (48%). Sawlogs are cut into sawn wood, while veneer logs are sliced into veneers and transformed into plywood. Pulpwood is processed into wood pulp and contributes to 40% of paper production, while the remaining 60% is sourced from recovered paper and other pulp. The remaining industrial roundwood is utilized as a wide range of wood products such as poles and piling. These processes generate wood chips, particles, residues, and wood fibre as intermediate products. These intermediates are further processed to produce wood-based panels, including particleboard, oriented strand board (OSB), and fibreboard, which are used directly in construction and furniture. Alongside, production of different wood products generates wastes that are burned for energy. The CHARM model applies similar relationships to future wood demand for wood products to estimate national wood harvest levels in future years.

Because some wood products use ‘wastes’ of other wood products, we trace these consumed products back to required wood harvest levels. Figure 2 shows our estimate of annual global flows of wood from harvest to ultimate use for 2010.

## Carbon costs and land use

**Fig. 3:**
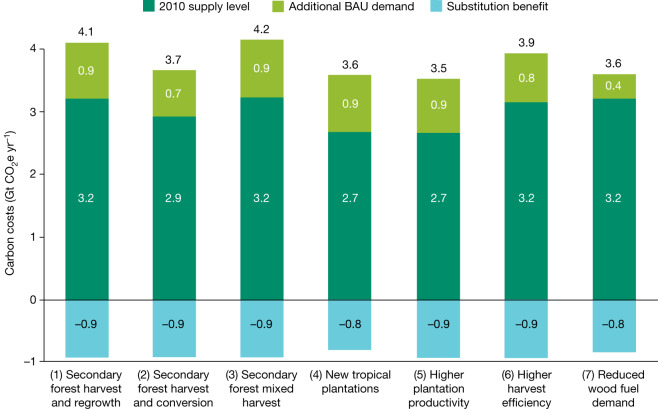
Estimated annual average carbon costs (Gt CO_2_e yr^−1^) of future global wood harvests between 2010 and 2050. Estimates of the average, annual, time-discounted carbon costs of global wood harvests under different scenarios of future wood supply. All scenarios (described in Table 1) assume the same future consumption except scenario 7, which cuts wood fuel use in half in 2050 from projected levels. Estimates of 3.6–4.2 Gt CO_2_e yr^−1^ apply a 4% discount rate to changes in carbon storage for 40 years after each year’s wood harvest. These account for forest regrowth and other changes in carbon storage pools over time but discount the value of those changes back to the year of harvest. They therefore represent harvest-year equivalent emissions—that is, the value of net changes in carbon each year if all occurred in the year of harvest (Extended Data Fig. 4 shows modest effects of varying discount rates from 2 to 6% or discounting over 100 years). Dark green regions represent emissions at 2010 wood supply levels, with light green representing emissions to meet  ‘additional business as usual (BAU) demand’. Blue represents estimated ‘substitution benefits’, which are estimated reductions in fossil fuel and other production emissions when using wood to replace concrete and steel in construction or as wood fuel to replace propane gas. Substitution benefits do not mean that wood use has climate benefits overall because they do not account for lost carbon storage—that is, biogenic emissions. Just as a small car still releases emissions even if less so than a large car, substitution benefits do not alter absolute emissions from wood harvest. Source: CHARM.

We next estimate the annualized carbon costs of global wood harvests from 2010 to 2050 under seven scenarios of future wood supply and demand (Table 1), discounting emissions and removals at 4% for 40 years after each wood harvest. We estimate costs at 3.5–4.2 Gt CO_2_e yr^−1^ (Fig. 3).

**Table 1:** Future wood supply scenarios analysed

Scenario	Assumptions
(1) Secondary forest harvest and regrowth	Wood first comes from existing plantations as of 2010; all other wood comes from middle-aged secondary forest; secondary forests regrow after harvest
(2) Secondary forest harvest and conversion	Wood first comes from existing plantation levels as of 2010; other wood initially comes from middle-aged secondary forests, which are converted to productive plantations that also provide wood in subsequent years
(3) Secondary forest mixed harvest	Same as scenario 1 except that half of secondary forests harvested are mature (40 years older than middle-aged) forests
(4) New tropical plantations	Same as scenario 1 except that 2 Mha of tropical agricultural lands are converted each year to plantations between 2010 and 2050; the non-harvest counterfactual assumes that land would regrow secondary forests
(5) High plantation productivity	Same as scenario 1 except that existing plantations increase in productivity by 25%
(6) Higher harvest efficiency	Same as scenario 1 except that tropical wood harvests of secondary forests increase in efficiency (reducing unharvested, felled wood) based on the estimated high-efficiency scenario in ref. ^28^
(7) Reduced wood fuel demand	Same as scenario 1 except that wood fuel consumption decreases in a linear pattern from 2010 to 2050 to reach 50% of 2050 baseline projections

**Extended Data Fig. 1:**
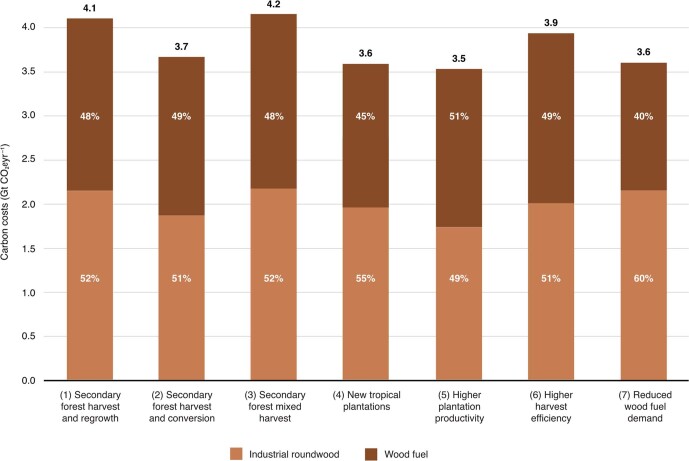
Share of annual carbon costs for 2010–2050 attributable to industrial roundwood versus wood fuel. Wood fuel is wood harvested deliberately for energy use. Industrial roundwood is wood harvested for initial use for other wood products although wastes of industrial roundwood are also burned for energy. This figure shows roughly equal emissions attributable to fuelwood and industrial roundwood except for Scenario 7, which reduces future use of fuelwood linearly toward 50% in 2050 relative to projected levels. Source: CHARM.

In general, existing levels of wood demand account for 78% of carbon costs between 2010 and 2050, with rising wood demand accounting for the remainder. Wood harvests at 2010 levels have costs of 3.2 Gt CO_2_e yr^−1^ in Scenario 1. On average, industrial wood and wood fuel each account for roughly half of carbon costs (Extended Data Fig. 1). The scenarios with lowest carbon costs have 50% lower wood fuel demand or 25% higher growth rates than existing plantations.

We also calculate a ‘substitution value’ based on estimated reductions in ‘production emissions’—for example, fossil fuel emissions when using wood in place of concrete and steel in construction or for traditional bioenergy. Substitution does not reduce emissions from wood harvest. However, just as a small car can emit less than a large car, substitution here means that the production emissions of wood can be less than substitute products. Despite variability in building and design and uncertainty in magnitude^39^, we use a mid-range substitution value from a comparative study^40^ for this substitution value for concrete and steel.

Under our assumptions, global substitution savings range from 0.8 to 0.9 Gt CO_2_e yr^−1^. Because this estimate ignores changes in forest carbon storage, it does not necessarily mean that wood use causes fewer emissions overall than concrete and steel or propane gas for cooking. If steel and concrete succeed in reducing their emissions over time, these substitution values could decline or disappear.

**Extended Data Fig. 2:**
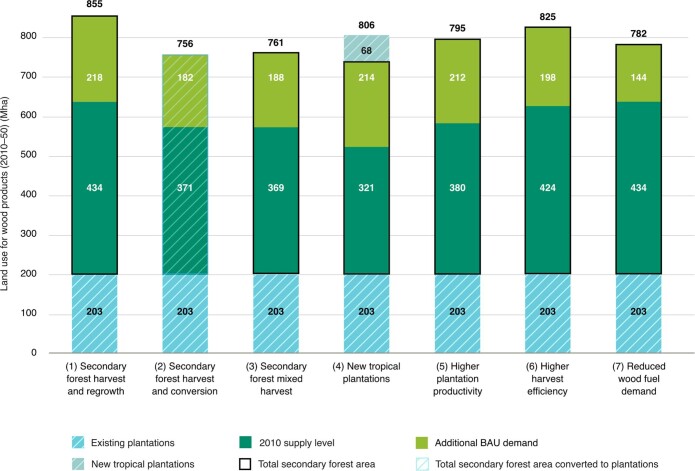
Land areas harvested 2010–2050 (clear-cut equivalents, million hectares). This figure shows estimates that wood harvests will need to take place on 761 to 855 million hectares over this 40-year period based on the scenario. It separates harvests required to meet 2010 consumption levels and those required to meet additional BAU (business as usual) demand. Clear-cut equivalents mean the land area if forests were harvested by clear-cut although with the efficiency estimated now for national wood harvests overall, which are often lower for selective harvest. Any hectares of plantations harvested more than once are counted only once. Source: CHARM.

We also estimate that 756–855 million hectares (Mha) of land would be harvested in the different scenarios using ‘clear-cut equivalents’, the area required if all wood were harvested by clear-cut (Extended Data Fig. 2). We use this measure because area and harvest levels of selectively harvested lands are mostly unknown. (Repeated harvests of a hectare of plantation count as one.) Relative to scenario 1, increasing plantation growth rates by 25% would reduce harvest area by 60 Mha and our linearly phased-in 50% cut in wood fuel would decrease harvest area by 70 Mha.

## Robustness of results

**Extended Data Fig. 3:**
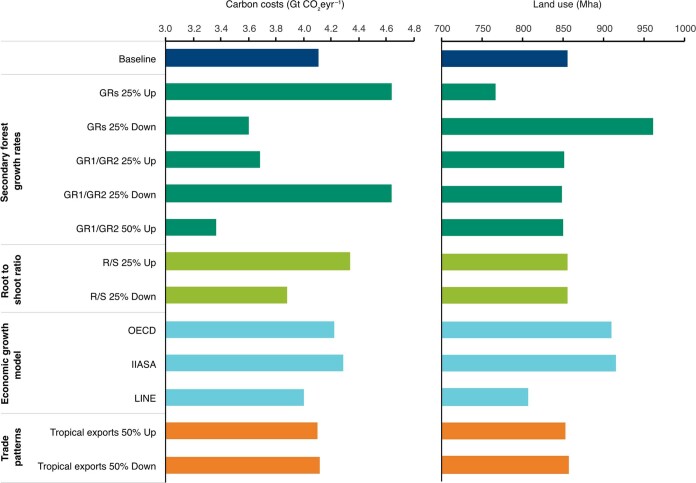
Sensitivity analyses of carbon emissions and land use change in scenario 1 in response to changes in growth rates, root-to-shoot ratios, per capita economic growth, and trade patterns. GR = growth rates. GR1 is young growth rate, GR2 is middle growth rate; both are used to parameterize a Monod function; GR1/GR2 up or down increases or decreases the ratio of early- to mid-age growth by the designated percentage; R/S = root to shoot ratio. OECD and IIASA provide different projected changes in GDP per capita by region and LINE estimates future growth based on continuation of past linear trend lines. Trade patterns vary the share of global exports provided from tropical forests (including plantations) relative to 2010 levels. See Supplementary Information for more description and tabular data. Source: CHARM.

Despite many uncertainties, sensitivity analyses of a range of model parameters support the robustness of our basic finding that forest harvests cause around 3–5 Gt CO_2_e yr^−1^ when focused on decadal effects (Extended Data Fig. 3).

Overall, results are probably conservative because they omit effects of harvests on soil carbon due to uncertain rates of loss and recovery. Nevertheless one meta-analysis found an average soil carbon loss after harvest of 11% within upper soil layers and evidence of more below^41^, and another found substantial losses when converting natural forests to plantations^42^. A worrisome study using advanced measurement techniques found large annual soil carbon losses in logged tropical forests in Borneo for many years after harvest (4.2 tC ha^−1^ yr^−1^)^43^. We also ignore indirect effects of forestry such as those triggered by road building, which have been estimated in the tropics at several times the direct effects^44^.

Equally transparent disclosures of input data and sources by subsequent researchers should lead to improved estimates over time. Estimates of biophysical, non-GHG effects of forest harvests could also change warming estimates although uncertainties continue to inhibit reliable estimates, particularly competing estimates of forest effects on cloud formation^45,46^.

## Insensitivity to discount rate

**Extended Data Fig. 4:**
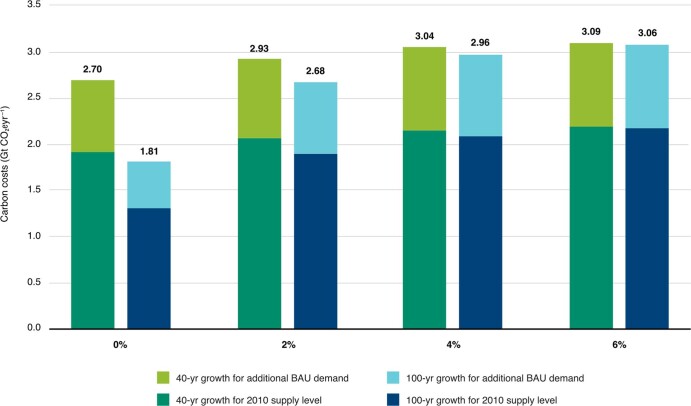
The effects of discount rate and years of growth on carbon costs of harvesting secondary forest in Scenario 1. This figure includes different carbon cost estimates of harvesting secondary forests not plantations in Scenario 1 varying discount rates from 0% to 6% and discounting over 40 or 100 years. Red (left) bars show discount effects of each year’s harvest on changes in carbon pools for 40 years; Green (right) bars discount these effects on changes for 100 years. Percentage changes are relatively modest except for applying a 0% discount rate over 100 years. These indicate similar valuing of the costs of wood harvest so long as there is even a modest social preference for earlier rather than later mitigation of emissions. Source: CHARM.

The use of small or large discount rates or extension of forest payback periods to 100 years has surprisingly modest effects. This is illustrated by the changed costs of secondary harvests in scenario 1 relative to our 4%/40-year central approach (Extended Data Fig. 4 and Extended Data Table 1). The 100-year payback at 4% reduces carbon cost by only 3%. Varying the discount rate from 2 to 6% even for 100 years varies costs by only −12% to +1%; even 0% discounting for 40 years reduces costs by only 11%. Large differences result only from 0% discounting over 100 years, which reduces annual carbon costs by 40%. These effects are modest because net emissions from wood harvest are spread somewhat evenly over early decades when discount rates matter most, and even a small discount rate sufficiently reduces the present discount value (PDV) of any net regrowth after 40 years that a higher rate makes little difference. Because our results change little even with a small discount rate over 100 years, they are robust if society has even a small preference for short-term over long-term mitigation.

## Meaning of economic effects

The counterfactual in our analysis is that forests not harvested would otherwise evolve independently of direct human activity: trees growing, dying, decaying simultaneously.

Some papers use economic models to attempt to construct a counterfactual that factors in the effects of wood demand on forest area or management^47^. Credible estimates of this type require large numbers of different types of supply-and-demand curves, which probably vary by country and almost none of which have been estimated econometrically using proper instruments. Global forest models also do not attempt to estimate how conversions of agricultural land to forests to meet wood demands in one location affect compensatory expansion of agricultural land elsewhere.

To some extent, however, our analysis is agnostic to these effects because our scenarios provide boundaries for possible future responses to economic forces. For example, scenario 2, which involves conversion of all harvested secondary forests to productive plantations, estimates the effect of extreme potential intensification. Scenario 4, which involves more tropical plantations, provides an ambitious example of increased forest plantings. No scenario reduces carbon costs below 3.5 Gt CO_2_e yr^−1^.

Regardless, even if reliable, estimates of other counterfactual alternatives due to economic forces would not alter estimates of the absolute carbon costs of wood harvests; rather, at best they compare harvest emissions with those from alternative human activities. For example, if a model claimed that without wood harvests more forests would be converted to crops, the analysis would compare carbon effects of one human activity, harvesting forests, with an alternative, more-emitting human activity, producing crops. Just as small cars still emit carbon even if less so than large cars, forest harvests would still emit carbon relative to no human activity even if less than cropping. Credible economic estimates of this comparison could have policy value, but understanding the absolute emissions from each activity, including forest harvests, would remain valuable. Among other reasons, by using this hypothetical, absolute estimates would indicate the carbon savings of reducing harvests with policies to block cropland expansion, a separate human activity.

## A potential mitigation option

Our estimates do not imply flawed estimates of increased atmospheric carbon but instead mean that ongoing, and probably increased, harvests of wood have major, although often ignored, carbon costs that should be attributed to human activity. Our estimated costs of 3.5–4.2 Gt CO_2_e yr^−1^ using 4% discounting are similar to common estimates for annual emissions from land-use change due to agricultural expansion of 3–4 Gt CO_2_e yr^−1^ (refs. ^10,48^).

These findings are, in a sense, good news because they imply that if people could reduce forest harvests, forest growth could do more to reduce atmospheric carbon, a potential mitigation ‘wedge’ that is rarely identified in climate strategies. As with other mitigation efforts, reductions have value only to the extent they do not shift emissions to another source. Over time, if more forests were able to mature this net sink would decline but these efforts would help ‘buy time’ for more climate mitigation activities to become viable.

## Methods

### CHARM basic structure

**Extended Data Fig. 5:**
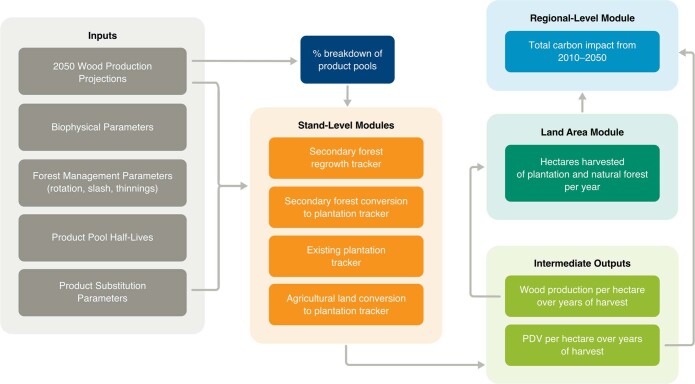
Schematic representation of the Carbon Harvest Model (CHARM) from inputs to outputs. The CHARM model has several basic modules shown in this graphic that translate projections for the consumption of different categories of wood products each year into land areas harvested and carbon impacts.

CHARM is a biophysical model developed for this paper and related work, which estimates the GHG consequences and landuse requirements to meet wood consumption levels. The principal version of the model runs in Python using input files from Excel. CHARM has components that include both stand level and global analysis (Extended Data Fig. 5).

Unlike other commonly used carbon ‘book-keeping’ models, which typically start with total wood harvest levels and therefore can be used only retroactively, CHARM uses estimates of four major wood product categories of consumption by country to estimate harvest levels. These wood product categories are: LLPs, which are essentially wood for construction and furniture; SLPs, which are paper and paperboard products; and VSLPs, comprising wood used immediately for bioenergy (VSLP–WFL); and very-short-lived products–industrial (VSLP–IND), which are wood wastes from the generation of other wood products that are burned for energy.

The model starts with existing wood sources and demands as of the year 2010. Demands for different wood products are aggregated into total wood demands by country. When estimating future production, the model assumes that existing global trade patterns remain the same. For example, if timber-importing countries increase their demand, the model assumes that imports will grow proportionately and that exporting countries will proportionately increase their exports to meet this increasing demand.

The model separates wood supplied by existing plantation forests and that supplied by secondary forests, each based on their harvest efficiencies and growth rates. Plantation forests are those we know are dedicated to wood production. Secondary forests, by definition, are forests that have been harvested and, given our rules on forest age for harvesting, are therefore more probably those involved in wood production. At the national and global level, the model uses information about each country’s forests and assumes that wood demand will first be met by plantations to the extent available in 2010 and that secondary forests will be harvested for the remainder. The model tracks the carbon consequences of harvesting these forests under allocation and regrowth management rules specified by the scenario.

Land requirements are defined as the area of plantation and of secondary forests harvested over a given period of focus, which is between 2010 and 2050 in this paper. The present version of the model uses an optimistic assumption that all forests harvested will be from secondary rather than from primary forests, which are typically more carbon-dense.

To estimate land-use requirements, the model assumes that all harvesting is achieved through at least small clear-cuts. (The model also allows for thinning of forests, but that is done on the same lands as those ultimately harvested and therefore does not increase harvest area counted.) The clear-cut assumption increases wood harvest per hectare and therefore reduces the area affected by harvest. In the tropics, although most non-plantation forest harvests occur selectively, there are problems of definition between selective harvests and miniature clear-cuts, as well as uncertainties about the quantities of wood removed by different logging techniques. These uncertainties make it challenging to provide a precise estimate of area affected. The area of land use calculated by CHARM should therefore be viewed as hectares of clear-cut equivalent (that is, the hectares that must be harvested assuming all hectares affected are clear-cut). However, estimated harvest efficiencies—that is, calculations of waste—are regionally based and therefore incorporate estimated losses from selective harvest where that is the predominant method, as in the tropics. The estimates are therefore clear-cut equivalents assuming harvest efficiencies at present levels.

These ratios between consumption and harvests by product category are then multiplied by the quantity of projected consumption for each year between 2010 and 2050 in each country, for each wood product category, to estimate harvest levels by country (factoring in trade). Conversely, the model allocates wood harvests within a country to different wood products based on estimates of their different product consumption levels.

Because of the questionable data quality of countries producing small quantities of wood, our global analysis estimated wood harvest in those 30 countries that produce 80% of the world’s wood and then divided that volume by 80% to generate a global estimate.

### Carbon costs and storage pools

To estimate GHG effects, CHARM globally applies an approach established for stand level analysis in the 1990s^34^ by tracking the flow of carbon between different carbon pools over time due to harvests. Any reduction in carbon stored in the aggregate of all pools from one year to the next means an emission by that quantity of that carbon to the atmosphere whereas any increase means a removal. The pools include live wood (including forest regrowth after harvest), forest residues, roots, wood in the different product categories and wood in landfills. For each hectare of forest in each year, the carbon cost is the difference between (1) the amount they would store without future harvests (non-harvest scenario) and (2) the quantity of carbon that forests and wood products would hold with future harvesting and planting (harvest scenario). A positive value means emissions and a negative value means carbon removal. This calculation therefore factors in both ongoing forest growth in a harvest if not harvested and forest regrowth after harvest.

The model assumes that harvested forests will be allowed to regrow. Even so, the model can differentiate between regrowth as a secondary forest or as a plantation. We note that an economic or behavioural model might seek to estimate the changed probability of growth or regrowth and could be valuable if reliable. However, in addition to the challenges of making these estimates (and their off-site consequences as well), this approach assumes that, if regrowth is stopped by another human activity, the emissions in the form of foregone sequestration should be assigned to that other activity and not to the harvest.

For the live vegetation pool, because clear-cuts are assumed, the pool is eliminated in the first year of harvest. However, this pool regrows over time according to growth rates specified for that forest type in each country. The live vegetation pool consists of above- and below-ground biomass pools. Below-ground biomass is estimated using a widely used power function for relating root to shoot biomass^49,50^. The model factors in dead wood remaining in the forest as a result of a harvest both in slash and roots, but it does not factor in changes in other downed dead wood. Forests typically have a layer of downed, dead wood not caused by harvest but due to dead trees and fallen branches. Although this pool may change over time, data are lacking—particularly across multiple forest types—of the changes in this forest pool as a result of harvest. In other words, the literature does not document whether forest harvests tend to expedite removal or degradation of already downed dead wood and, if so, how rapidly any such pool of carbon recovers with regrowth. (Estimates of dead wood stocks in the forest do not typically distinguish between those caused by a harvest, which CHARM does estimate, and those not caused by a harvest.) CHARM therefore assumes that this source of downed, dead wood is unaffected by harvest, is the same in both harvest and non-harvest scenarios and therefore does not need to be counted to determine the effects of wood harvest.

The model assumes that all VSLPs are burned and counted as an immediate emission, all SLPs are burned after use and that LLPs go to landfills as they decay. Meanwhile, the landfill pool can be interpreted as temporary storage because the carbon in wood products is not immediately released into the atmosphere. However, some percentage of the carbon emitted from the landfill is converted to methane, which has a much higher global warming potential and is counted as carbon dioxide equivalents based on its global warming potential over 100 years.

**Extended Data Fig. 6:**
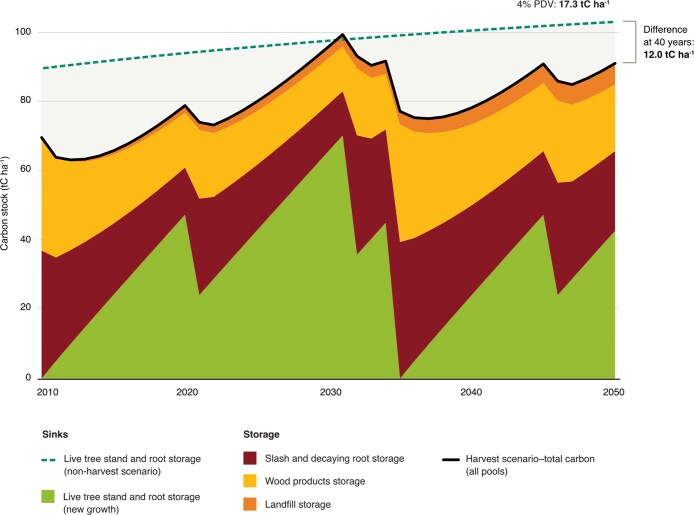
Changes in carbon pools over 40 years due to conversion of Southeastern U.S. average secondary forest to loblolly pine. This figure illustrates the changes in carbon pools estimated by the model for each wood harvest using one forest type as an example. PDV = present discount value. The dotted green line shows carbon storage without forest harvest. The difference at 40 years shows the effect on carbon storage 40 years later, which is equivalent to zero discounting. The PDV value shows the value of changes in carbon over time that would be equivalent to these changes if they all occurred at the year of harvest, based on discounting these changes, i.e., both emissions to the atmosphere and removals from the atmosphere, by 4% per year. In this example, the substitution value is not factored into the PDV. Source: CHARM.

Extended Data Fig. 6 shows the changes in carbon storage for loblolly pine plantations in the Southeastern United States. In the first year of harvest there is a net increase in carbon emissions (represented by the vertical difference between the dotted green line and solid black line). In the second year there are further emissions as some of the felled wood decays or is burned, which can be seen by an expanding distance between the two lines. In the later years of each harvest cycle, due to more rapid forest regrowth the black and dotted green lines converge, representing net removals of carbon from the air.

### Wood consumption, harvesting and trade data

Model development needed extensive effort to estimate the quantities of wood harvests required to meet each unit of wood production consumption by wood product category in a manner consistent with FAO consumption, production and trade data. Estimation of relationships between production and consumption data presents challenges because FAOSTAT gathers and reports wood product consumption and harvests in different types of units (such as weight versus volume and in products that have different, although unstated, water contents and therefore shares of dry matter). FAOSTAT also reports intermediate wood products between harvests and final consumption, the production of which generates significant wastes, some of which are then used for other products whereas others are typically burned. We used information from a variety of sources including unit conversion estimates, FAOSTAT estimates of standard waste levels in pulpwood production and estimates of sawn wood wastes implied by production data and which therefore varied by country. Trade data were also of inconsistent quality and, for some countries, implied physically impossible or highly unlikely consumption:production ratios. We developed rules to address data inconsistencies and data quality. The estimation methods used are described in further detail in the Supplementary Information.

### Biophysical model inputs

The model uses a variety of biophysical inputs. One is secondary forest growth rate over time, which has consequences both for the forest if not harvested and for regrowth after harvest. There are many uncertainties about growth rates, and changes in growth rates over time, over large forest areas. Even different forest types in the same compact area can have highly varying growth rates and patterns^51^, and efforts to identify even dominant forest types spatially tend to have high error rates^52^. Our global model derives growth rates for secondary forests from Harris et al.^25^, which uses a variety of sources discussed in that paper and its supplements. We supplemented this information with further data on the relationship between young and middle-aged secondary forest growth rates^49^. Similar to default guidance from the Intergovernmental Panel on Climate Change for national GHG reporting, Harris et al.^25^ estimate growth rates in broad time bands, one forest growth rate for younger forests of less than 20 years and another for more than 20 years. Because changes in time matter more to our model than to the estimates in that paper, we used these time bands to derive continuous growth rates using a Monod function found to be a reasonable proxy for forest growth rates in general^53,54^.

For plantations we first applied the growth rates from Harris et al.^25^ to the countries available in the Spatial Database of Planted Trees (SDPT v.1.0) and then supplemented the boreal and some EU countries, such as Canada and Russia, with average secondary forest growth rates. We also compiled data from a variety of literature and national reports for important timber producers such as Brazil, China, Indonesia and the United States, which are described in Supplementary Information.

Other inputs to the model for each forest type include root:shoot ratios, the portion of above-ground biomass left behind after harvest (slash rates), the proportion of above-ground biomass removed during thinnings and the rotation period. The proportion of carbon in harvested wood allocated to each product pool derives from the estimated consumption share of that product in that country. The model also requires decay rates for each carbon pool and inputs for allocation of that carbon to different subsequent pools (for example, landfills).

All input values and their sources, and further details regarding the Monod functions, are described in Supplementary Information Section 3. Supplementary Table 5 provides weighted average national forest growth parameters in the 30 countries used for this analysis. Supplementary Table 6 supplies the plantation rotation periods used and information sources. Supplementary Table 7 gives secondary and plantation slash rates by country. Supplementary Table 8 describes the half-lives used for the ‘decay’ of carbon in different carbon pools. The uncertainties of secondary forest growth rates and root:shoot ratio are discussed in Supplementary Information Section 5.

### Production emissions and substitution values

The generation of wood products also releases fossil emissions and potentially trace gases in planting, harvesting and the production process. Because there are numerous data uncertainties on a global basis about how much fossil energy is used in harvesting wood and producing wood products, CHARM does not at this time incorporate these emissions.

Although comparisons between emissions from the use of wood products and alternative non-wood products do not reduce the absolute emissions from use of wood products, there is keen interest in whether wood use has lower emissions than alternatives. A full calculation of this requires calculation of the effects on biogenic carbon as well as production emissions. Even so, and because CHARM separately calculates biogenic emissions, CHARM is now programmed to estimate potential ‘substitution’ savings in production emissions when using wood to replace concrete and steel in construction. Estimates vary substantially owing to the different quantities of each material required for different buildings and different construction methods. Our calculation uses a central value from a review of other studies^40^ of 1.2 tonnes carbon saved from production processes for each 1 tonne of carbon in wood used in construction that substitutes for concrete and steel. The benefit also depends on the share of harvested wood used in construction. As described in the Supplementary Information, we used estimates by country from Zhang et al.^55^.

CHARM also estimates substitution benefits from the use of traditional firewood and charcoal in place of fossil fuels. Assuming that the alternative would be the use of propane gas, we use a substitution factor of 0.175 tonnes of carbon saved from avoided fossil fuel use for each 1 tonne of carbon from wood. This is based on estimates of relative energy output, charcoal and firewood production efficiencies and stove output and use efficiencies provided by the lead author of ref. ^31^.

### Factoring time into carbon calculations

In addition to estimation of physical changes in emissions and removals of GHGs to the atmosphere over time due to each year’s wood harvests, the model estimates the value of these changes in the year of harvest using different discount rates. When the model uses a zero discount rate it estimates the physical change in atmospheric carbon after the period analysed, which can be 40 or 100 years after each harvest. In effect, a zero discount rate assumes that the change in atmospheric carbon at the end of the period is of equal value to this same change in carbon if it occurred in the year of harvest.

Discounting assigns a higher value to earlier emissions reductions. The model expresses carbon emissions as a value but based on an equivalency to emissions that occur only in the year of harvest—that is, harvest-year equivalent emissions. This form of valuation establishes a relationship between the value of emissions or mitigation in different years but does not need to specify an absolute dollar value for each tonne of carbon, which could be separately debated and determined.

The choice of a discount rate is a policy decision, which can represent two benefits of earlier mitigation. One benefit is to recognize the value of immediate reductions to avoid both intermediate and permanent damages from rising temperature (for example, the effects of ice sheet melting or biodiversity loss) and to postpone the date of crossing a variety of climate thresholds. Earlier mitigation in effect holds down damages immediately and increases the time in which people can improve technology and organize the political will and resources to combat climate change before crossing thresholds.

The other benefit of earlier mitigation results from the time value of money. Applying a 4% discount rate in effect assigns a 4% rental charge each year to additional carbon in the atmosphere. That equals the price of borrowing money at a commonly estimated long-term cost of capital to pay another person to mitigate emissions to compensate. As discussed in ref. ^33^ in the context of land-use conversion, this discount rate also generates results consistent with the amortization period used for land conversion in US bioenergy policy.

The value discounted in each year after each harvest back to the year of harvest is the change in atmospheric carbon that year—that is, the difference between emission (or removal) in that year and that in previous year. This formula in the year of harvest *h* (for example, 2010) is:

1 $${\rm{PD}}{{\rm{V}}}_{h}=\mathop{\sum }\limits_{t=0}^{N}\frac{\Delta {C}_{{\rm{change}},t}}{{\left(1+d\right)}^{t}}$$

where *t* is the number of years since harvest in year *h*, *d* is the discount rate (4%), *N* is the number of years for growth since harvest in the scenario (for example, 40 or 100 years) and *C*_change,*t*_ is the change in emissions (or removals) in the year *t*. Extended Data Table 2 shows the calculation of time discounting of 4% over 40 years for a plantation conversion scenario shown in Extended Data Fig. 6.

Present discount value is calculated identically for each subsequent year of harvest. The cumulative PDV of emissions between 2010 and 2050 is the sum of these carbon costs over 40 years, and they therefore do not represent the carbon added to the atmosphere in 2050 by forest harvests between 2010 and 2050. That alternative quantity of carbon would be larger because it would not factor in the full 40-year regrowth of forests harvested after 2010. However, this method in effect assigns a discounted value for projected forest regrowth regardless of which year the harvest occurs—for example, even in the year 2049.

For national and global results we then determine the total discounted carbon cost in year *t* by multiplying the PDVs of each hectare by the number of hectares harvested of that same forest type in the year harvested. This is done separately for both plantations and secondary forests, producing the formula:

2 $${{\rm{PDV}}}_{{\rm{total}}}=\mathop{\sum }\limits_{h=2010}^{K}{\rm{PD}}{{\rm{V}}}_{{\rm{secondary}},h}\times {a}_{{\rm{secondary}},h}+\mathop{\sum }\limits_{h=2010}^{K}{\rm{PD}}{{\rm{V}}}_{{\rm{plantation}},h}\times {a}_{{\rm{plantation}},h}$$

where *h* represents the year of harvest starting from 2010, *K* represents the number of years of harvests (for example, 40 years) and *a* represents the new area of one forest type harvested in year *h*. The next subsection describes the calculation of area required for each forest type.

### Land area calculation

Due to the unknown levels and quantities of wood removed from selective harvests, CHARM calculates the area of land use as hectares of clear-cut equivalent (that is, the hectares that must be harvested assuming all hectares affected are clear-cut). This assumption increases wood harvest per hectare relative to selective harvests and therefore reduces the estimate of area affected by harvest. This method is used because of inadequate data available for the quantity of wood harvested through clear-cuts and that through selective harvest. This calculation of land requirements reflects the quantity of wood generated per hectare at the estimated efficiencies by country. The quantity of wood required is also based on the ratio of each category of wood production consumption to harvest levels required to generate that level of consumption—that is, it factors in wastes. For the period 2010–2050 the model assumes linear growth in consumption for each product category from 2010 to 2050. Plantation areas are harvested first, and secondary forests are harvested as needed to supply the remaining quantities of wood.

### Projection of 2050 wood demand

To project future wood demand, CHARM starts with 2010 consumption levels (calculated as an average of 2006–2014 consumption) by country for consumption and production of different wood products and harvest levels, using data from FAOSTAT^56^ after a system of quality controls (Supplementary Table 1). For each country and year, we first calculated net exports by subtracting imports from exports. Future projections assume that, within each country the share of consumption supplied by net imports will remain the same as in the base year and that each country will provide the same share of aggregate global exports.

To estimate future wood product demand by country, we use a log-transformed fixed-effects model^57^ and project wood demand for each country and each product category. The fixed-effects model applies the same relationship of wood consumption to each country’s per capita income growth but starts with each country’s initial wood consumption. We separated countries into developed and developing to avoid overestimation of future wood consumption in high-income countries. For wood products consumption—and based on available data—we selected sawn wood and wood-based panels to represent all LLP, paper and paperboard to represent SLP and wood fuel to represent VSLP-WFL. The historical socioeconomic statistics include GDP and population from the World Bank for 1961–2020^58^. We used projected growth percentages between 2010 and 2050 for GDP per capita and population based on from average GDP growth prediction from three sources: the Organisation for Economic Co-operation and Development (‘middle of the road’)^59^, the International Institute for Applied Systems Analysis model SSP2 scenario^60^ and a linear trend line that we calculated for the period 1991–2010. Predictor (independent) variables in the fixed-effects model include population, GDP per capita and the year after 2000, which serves as a proxy for technological and policy changes since 2000, when the internet usage boom started and modified subsequent paper requirements. The fixed-effect model establishes 12 relationships (‘models’) based on three different types of wood product, two different trend lines in developed and developing countries and two different regression formulae, one using our time variable and one without. All models have high *R*^2^ full (over 0.88) and significant *P* values (over 0.05) and have a residual standard error between 0.32 and 0.84 (Supplementary Table 2). We apply the coefficients (Supplementary Table 3) of predictor variables to independently estimated changes in future populations and GDP by country, and use the resulting estimated consumption levels to force the model. The Supplementary Information provides statistics for model fits and further information about the fixed-effects model and application. Extended Data Table 3 shows the consumption of different wood products by country in 2010 and as projected for 2050. Supplementary Table 4 shows a comparison of our projections with those of other studies.

## Online content

Any methods, additional references, Nature Portfolio reporting summaries, source data, extended data, supplementary information, acknowledgements, peer review information; details of author contributions and competing interests; and statements of data and code availability are available at 10.1038/s41586-023-06187-1.

## Supplementary information

Supplementary Information This file includes a single PDF file containing Supplementary Methods,  Supplementary Tables 1–15, List of abbreviations, and Supplementary References. 

## Figures and Tables

**Extended Data Table 1:**
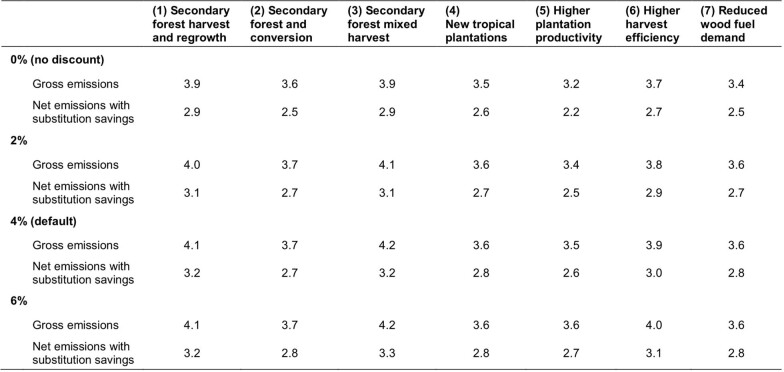
Sensitivity analysis – Annual average time-discounted carbon costs (Gt CO_2_e yr^−1^) of global forestry at different discount rates over 40 years

This table shows modest effects of varying discount rates from 0% to 6% over 40 years. For example, in Scenario 1, the difference is only between 3.9 and 4.1 Gt CO_2_e y^−1^.

**Extended Data Table 2:**
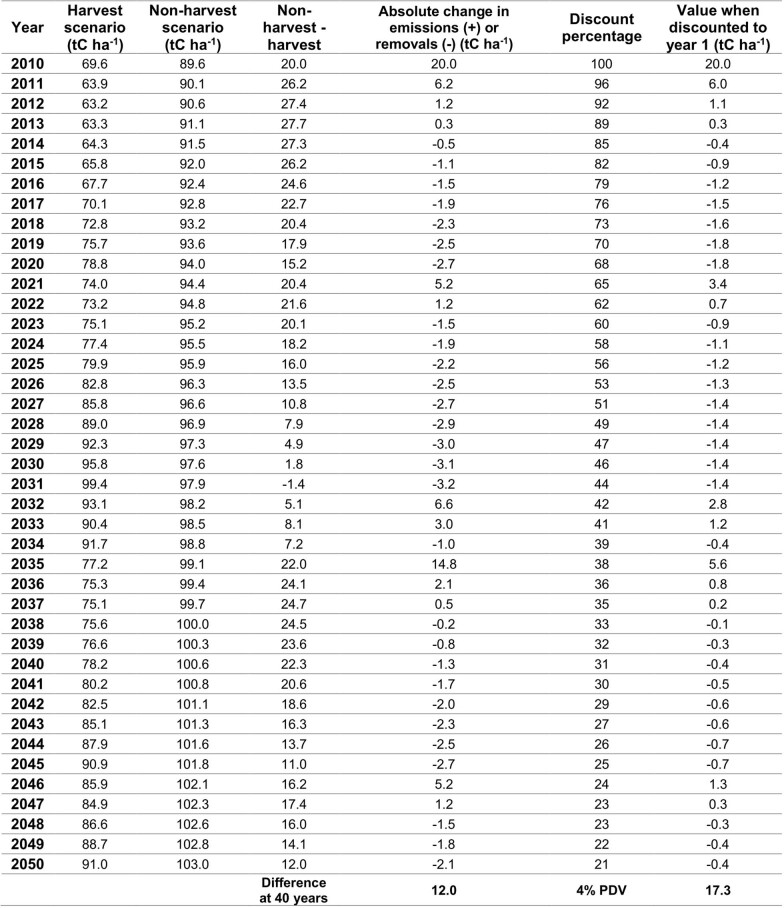
Illustrative example of time discounting calculation (4% for 40 years post-harvest) of one hectare of Southeastern U.S. secondary forest with conversion to loblolly pine plantation

In this example, wood is assigned to wood products based on existing usage and without substitution effect. The absolute carbon change 40 years after harvest is 12.0 tC ha^−1^ (summing the “absolute change” column) and the PDV is 17.3 tC ha^−1^ (summing the “value when discounted to year 1”).

**Extended Data Table 3:**
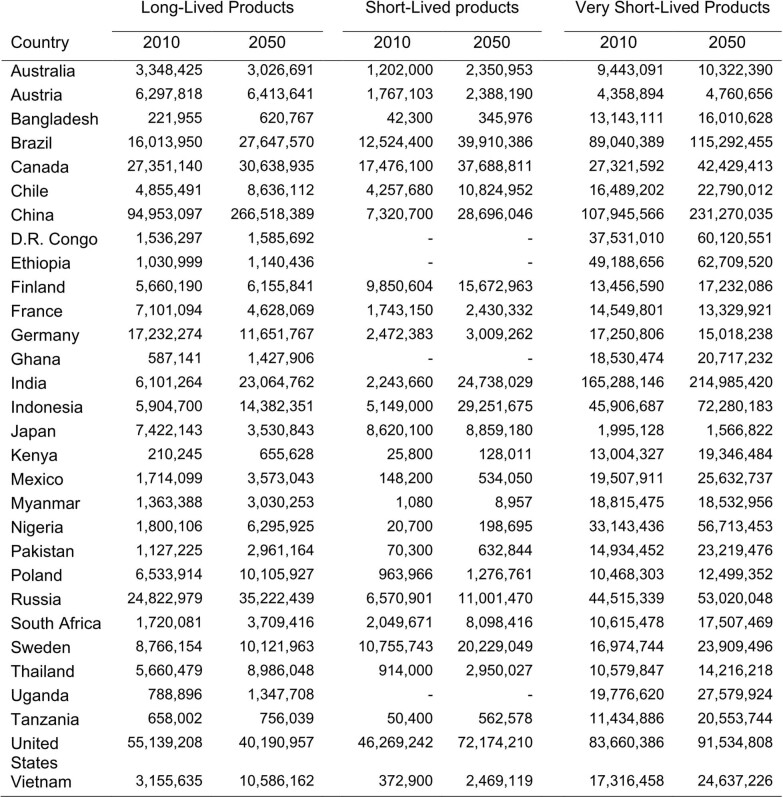
Current and projected future wood products production (tonnes dry matter) in the 30 countries used by CHARM Model

This table shows estimates of future production by wood product type by each of the 30 countries used for CHARM. Projections in 2050 assume the same share of trade for each wood product category as in 2010.

## Data Availability

Most input data for the CHARM model are described in the Supplementary Information. FAOSTAT production and trade data on forestry products at the national level are available from https://www.fao.org/faostat/en/#data/FO. Historical GDP and population data at the national level are available from https://data.worldbank.org/indicator/NY.GDP.PCAP.CD and https://population.un.org/wpp/Download/Standard/CSV/. Future socioeconomic scenarios are available from https://tntcat.iiasa.ac.at/SspDb and using data from https://data.worldbank.org/indicator/NY.GDP.PCAP.CD. Any other data used in this study are contained within input files available with the code for the CHARM model as described below.

## Code availability

Input and output data at the national level for the CHARM model that support the findings of this study are available along with the model code (https://github.com/wri/charm-global-level).
