# Supplementary material for: The carbon costs of global wood harvests
Source: Nature. 2023 Jul 5;620(7972):110–5. doi: 10.1038/s41586-023-06187-1 (PMC10396961; doi:10.1038/s41586-023-06187-1)
Supplement: Supplementary file 1 — This file includes a single PDF file containing Supplementary Methods, Supplementary Tables 1–15, List of abbreviations, and Supplementary References. [file 41586_2023_6187_MOESM1_ESM.pdf]

nature portfolio

https://doi.org/10.1038/s41586-023-06187-1

## Supplementary information

# The carbon costs of global wood harvests

In the format provided by the
authors and unedited

# Supplementary Information for
# The Carbon Costs of Global Wood Harvests

Liqing Peng[1*], Timothy D. Searchinger[1,2*], Jessica Zionts[1], Richard Waite[1]

[1] World Resources Institute, Washington, DC, USA.

[2] School of Public and International Affairs, Princeton University, Princeton, NJ, USA.

**Correspondence to**: Timothy D. Searchinger (tsearchi@princeton.edu); Liqing Peng (liqing.peng@wri.org)

## Table of Contents

A. Supplementary Methods ........................................ 2
1. Wood consumption ........................................ 2
 1.1 Wood production and consumption database for 1961-2020 ........................................ 2
 1.2 Projecting future wood demand ........................................ 6
2. Wood harvest and land use ........................................ 11
3. Biophysical Model Inputs ........................................ 13
 3.1 Secondary forest growth function and parameters ........................................ 14
 3.2 Existing plantation forest growth rates ........................................ 14
 3.3 Harvest parameters ........................................ 17
 3.4 Decay parameters ........................................ 18
4. Model Scenarios ........................................ 18
 4.1 Global wood supply scenarios ........................................ 18
 4.2 Harvest and non-harvest growth rates ........................................ 20
5. Sensitivity Analyses ........................................ 20
 5.1 Growth rates ........................................ 20
 5.2 Root-to-shoot ratio ........................................ 20
 5.3 Economic growth model ........................................ 21
 5.4 Trade patterns ........................................ 21
B. Supplementary Tables ........................................ 22
C. List of Abbreviations ........................................ 33
D. Supporting Information References ........................................ 35

# A. Supplementary Methods

## 1. Wood consumption

### 1.1 Wood production and consumption database for 1961-2020

**FAOSTAT data**

This section describes the way we estimated the flow from wood harvests to wood products, as presented in Figure 2. Because the FAOSTAT[1] is the ultimate source of data on both wood consumption and harvest levels globally, the model must develop these relationships in a manner consistent with FAOSTAT data. However, the FAOSTAT independently reports data on various wood product consumption, trade, and harvest levels, using different units and without explicitly accounting for physical transformations between raw, intermediate, and finished products. Many wood product categories also overlap. Some data are missing, and some are not credible. For example, trade data regarding different products is often incomplete and some countries have reported data for harvest, production and trade that are physically impossible. A major developmental feature of CHARM was developing the relationships between wood product consumption, trade, and harvest levels in a manner consistent with these different FAOSTAT data products, and accounting for data availability and to some extent quality.

Global roundwood harvests in FAOSTAT are divided into two major categories: industrial roundwood (FAOSTAT item code 1865) and wood fuel (1864). Industrial roundwood itself falls into three categories: generally larger logs that are sawn into timber or peeled to provide veneer, typically called “sawlogs and veneer logs” (1868); generally smaller logs harvested for paper, particleboard, and paperboard (e.g., cardboard), called “pulpwood” (1870 and 2038); and “other industrial roundwood” (1871) that are used for such uses as poles, fencing, wood wool, tanning, and distillation. FAOSTAT always reports the production quantities for the above categories, but not all of them have import/export quantities reported. Most of the time, only the two major categories, industrial roundwood and wood fuel, have both production import/export quantities.

The wood harvests provide the raw materials for manufactured forest products. Sawlogs and veneer logs are processed in sawmills and are then turned into sawn wood (1872) and plywood (1640). The production of sawlogs generates wood chips and particles and wood residues (1619), some of which are used for particleboard (1697), OSB (1606) and fiberboard (1874) and some of which are used for pulp production or are burned for energy. Pulpwood is primarily used for wood pulp (1875), and some of it is also used for particleboard and fiberboard. Wood pulp comes from pulpwood and wood residues from sawlogs, and it is used for 40% of the raw materials for paper and paperboard (1876). The remaining 60% is from recovered paper (1669) and other pulp (1668). In other words, both sawlogs and pulpwood can be used for particleboard, OSB, fiberboard, and wood pulp. Wood-based panels (1873), a commonly used aggregated primary forest product, are the sum of particleboard, OSB, fiberboard, and plywood. Wood chips and particles and wood residues (1619) exclude the chips in the production of pulp, particleboard, fiberboard, as well as chips counted as pulpwood, wood fuel, and other industrial roundwood. In summary, sawlogs, veneer logs, and pulpwood are turned into sawn wood

(SNW), wood-based panels (WBP), and wood pulp (WPL). We define these as main industrial roundwood (IND-M) products. Industrial roundwood (IND) is the sum of main industrial roundwood and other industrial roundwood (IND-O).

Supplementary Table 1 lists the main FAOSTAT items we use to calculate wood demand (consumption). In country $N$ ($N = 1 \ldots 176$) at year $T$, we first calculated net exports by subtracting imports from exports. If exports or imports are missing from the data for a country, then net exports are set to "missing" and not counted. We then calculated consumption by subtracting the net exports from production. If both production and net exports are missing, consumption is set to "missing" and is not counted. If either production or net exports is missing, consumption is set to "production" or "net imports", assuming the missing element is gap filled by zero.

**Wood balance and waste**

Closing the material balance using FAOSTAT requires three preprocessing steps. We first converted the units when in solid volume or weight to cubic meters. The unit of wood pulp or paper is converted from metric tons (10% moisture content) to cubic meter using a conversion factor (= 1.87 $m^3\ t^{-1}$):

$$CF = \frac{1 - MC_w}{\rho_b} \quad (1)$$

$MC_w$ is the 10 percent moisture content and $\rho_b$ is the global average wood basic density 0.48 ton $m^{-3}$ derived from the FAOSTAT forestry products conversion guideline. Second, we identified if there is missing data in other industrial roundwood, then we calculated other industrial roundwood using industrial roundwood minus the sum of sawlogs and veneer logs and pulpwood. If other industrial roundwood and either sawlogs and veneer logs or pulpwood are missing, then other industrial roundwood is set to zero. Third, we implemented two tests of data quality for industrial roundwood at the country level. If country data in a specific year does not pass either of the following criteria, we set the records as missing for all industrial roundwood products for this year in this country: industrial roundwood supply and the consumption of wood products (sawn wood, wood-based panels, wood pulp) should be positive and/or total sawlogs domestic use (production minus net exports) should be greater than sawn wood production. Last, we set the quantity elements (production, consumption, net exports) for paper and paperboard or wood fuel as missing if its consumption is negative.

Wood products require much more roundwood than the actual quantity of the products. The production of industrial roundwood such as pulping and sawing, generates wood waste. Determining the amount of industrial waste is important for estimating the immediate carbon emissions for burning. We first checked reported conversion factors, such as the input-to-output ratio and the yield. The pulp yield is fairly stable. Based on the FAOSTAT forestry products conversion guideline[2], the global average input-to-output ratio for pulp is 3.58 $m^3\ t^{-1}$, and the pulp weight to solid volume conversion factor is 1.87 $m^3\ ton^{-1}$ (see above). The pulp waste in solid volume per ton of wood pulp becomes 3.58 – 1.87 = 1.71 $m^3\ t^{-1}$, so the waste to roundwood

percentage is 1.71 / 3.58 = 48%. In other words, around half of roundwood devoted to wood pulp is burned for energy use as waste. Similarly, the global average input-to-output ratio of other industrial roundwood is 1.4 $m^3$ $m^{-3}$, which means about 29% of the other industrial roundwood is wasted.

For sawn waste, there is not enough reliable information to use that data directly from another source. We therefore developed a material balance approach to estimate the global and national industrial waste from pulping and sawing. Although FAOSTAT does not provide the data directly, we can derive them because the industrial roundwood domestic use (production plus imports minus exports) should be balanced by the sum of the production of sawn wood, wood-based panels, wood pulp, other industrial roundwood, the pulp waste that is estimated above, and sawn waste (Figure 2).

We calculated the actual pulp and sawn (PS) waste ratio in each country using Equation 2:

$$\text{PS waste ratio} = \frac{C_{IND} - P_{IND\text{-}O} - P_{SNW} - P_{WBP} - P_{WPL}}{C_{IND\text{-}O} - P_{IND\text{-}O}} = 1 - \frac{IND\text{-}M}{IND\text{-}PS} \quad (2)$$

where $C_{IND}$ is the domestic use of industrial roundwood (production plus imports and minus exports), $P_{IND\text{-}O}$ is the production of other industrial roundwood, and $C_{IND} - P_{IND\text{-}O}$ is defined as industrial roundwood used for pulping and sawing (*IND-PS*). $P_{SNW}$ and $P_{WBP}$ and $P_{WPL}$ are the production of sawn wood, wood-based panels, and wood pulp, and the sum of the three is defined as the main industrial roundwood product (*IND-M*). We gathered all the records during the baseline period from 176 countries that have valid records and then derive the distribution of the PS waste ratio. We observed an average at about 48% between 2006 and 2014, and a standard deviation at about 22%. This estimate allows us to define hard boundaries for the waste percentage in each country.

We set a waste ratio minimum (10%) and maximum (70%) to determine whether a country has an excessive surplus or excessive deficit of industrial roundwood supply, which is likely the result of inaccurate wood accounting. If the PS waste ratio is negative (such as in China and Japan), the country does not have enough industrial roundwood supply. If the PS waste ratio is less than 10%, the efficiency is too high to be true. If the PS waste ratio is greater than 70%, the country may have excessive industrial roundwood supply, as the efficiency is too low. To adjust these unrealistic country-level imbalances, we capped the PS waste ratio to between 0.1 and 0.7. Therefore, we can obtain the adjusted IND-PS' using Equation 3:

$$\text{IND-PS}' = \begin{cases} \frac{IND\text{-}M}{1-0.7}, \text{PS waste ratio} > 0.7 \\ C_{IND} - P_{IND\text{-}O}, \quad 0.1 \le \text{PS waste ratio} \le 0.7, \\ \frac{IND\text{-}M}{1-0.1}, \text{PS waste ratio} < 0.1 \end{cases} \quad (3)$$

When the PS waste ratio is less than 0.1, IND-PS' – IND-PS is defined as the additional consumption (additional production or imports) required for the country. When the PS waste ratio is greater than 0.7, IND-PS' – IND-PS is defined as the reduced consumption (additional exports) for the country to supply the need from the countries without deficits. There are three groups of countries regarding their waste ratios: Group 1 has reasonable waste (no adjustment needed, IND-PS' – IND-PS = 0), Group 2 has too much waste (IND-PS' – IND-PS < 0), and Group 3 does not have enough waste (IND-PS' – IND-PS > 0).

The first step is to adjust the net exports of the net importer countries (net exports < 0). For countries (e.g., China, Japan) that do not have enough waste (IND-PS' – IND-PS > 0), net exports will increase by the additional waste (IND-PS' – IND-PS). For countries (e.g., India) that have too much waste (IND-PS' – IND-PS < 0), we removed the extra waste from the net exports (IND-PS' – IND-PS < 0). After the first step, we calculated the world total industrial roundwood net exports, which need to be balanced by the exports from the net exporter countries (net exports > 0). We then updated the national net exports and redefine the net importer and net exporter countries.

The second step is to adjust the net exporter countries (net exports > 0). The first goal is to meet the world total industrial roundwood net exports by adjusting the net exports in the three groups. The second goal is to recalculate the PS waste ratio in Groups 2 and 3 by adjusting the (production – net exports). We assume that Group 3's net exports should not increase because they already have a wood deficit. Therefore, to adjust the PS waste ratio, we only change their production. (Production – net exports) of Group 2 should reduce, so net exports must increase, and production may change or may not change. Group 1's PS waste ratios remain the same; therefore, Group 1's net exports and production should increase at the same quantity. We calculated the total net exports in Groups 1 and 2 and then calculated the net export share among these countries. The shares of net exporter countries are then used to scale their net exports to meet the world total industrial roundwood net exports. After that, the PS waste ratios of the three groups range from 0.1 to 0.7.

**Relationships between wood product consumption and harvest levels**

The above procedures create an adjusted FAOSTAT database for the nine-year period of 2006–14 that has reasonable national PS waste ratios and consistent production and consumption numbers. CHARM determines emissions based on the half-lives of wood products. Therefore, we define three major categories as inputs for CHARM: LLPs, which are uses of wood for construction and furniture and other long-term uses; SLPs, which are various paper products; and VSLPs, which are essentially uses of wood for energy. The LLP category includes solid wood products such as sawn wood, wood-based panels, and other industrial roundwood uses (IND-O, about 71% of other industrial roundwood). The SLP category consists only of wood pulp, which is directly related to pulpwood or sawlog wood harvests. The VSLP category includes two subcategories: wood fuel (VSLP-WFL) and industrial waste (VSLP-IND). Industrial waste (VSLP-IND) also includes two groups: pulp and sawn waste and other industrial roundwood waste (VSLP-IND-O, about 29% of other industrial roundwood). For 2010 reference year, we

calculate the national averages for LLPs, SLPs, and VSLPs in cubic meters. Each one has production, net exports, and consumption. They can be converted to dry matter tons by multiplying the global average wood basic density 0.48 t $m^{-3}$.

## 1.2 Projecting future wood demand

### Fixed effects model

Future wood harvests are based on projections of future world wood demand. Wood harvesting has been rising, driven by increased consumption. A preliminary regression analysis shows that industrial roundwood consumption generally has significant positive relationships with GDP per capita. However, wood consumption varies with socioeconomic factors (e.g., demographics, income levels, technology), and also varies significantly between countries, apparently influenced by the availability of wood. For example, countries such as Sweden and the United States, which have abundant forests, use far more wood than many other countries, which have few forests. We therefore used a fixed effects (FE) model[3] and reported the projections of wood demand for each country, each product category, and each scenario from 2010 to 2050.

Trend lines of wood consumption implicitly factor in relationships between demand and supply because all those demand and supply interactions were occurring in the past. The FE model applies the same relationship of wood consumption to each country's per capita income growth but starts with each country's initial wood consumption. The FE model helps represent the persistent differences that are caused by the specific properties in the countries, such as natural forest area, and are not related to the GDP per capita. Extrapolating the trend lines to the future has the disadvantage of assuming the future will be the same as the past and ignoring other factors that might change demand for any one type of product. However, this is the best guess because the past relationships (parameters) between wood demand and its drivers are not clearly known, and even if they were, these relationships can also change in the future.

For our projection of wood products consumption, we selected sawnwood (SNW), wood-based panels (WBP), paper and paperboard (PPB), and wood fuel (WFL). This is because their consumptions are directly driven by socioeconomic factors and have statistics that can be tracked through trade. (Items such as wood pulp, other industrial roundwood, and industrial waste do not have trade statistics.)

The historical socioeconomic statistics include GDP and population from the World Bank for 1961–2020. We use projected growth percentages between 2010 and 2050 for GDP per capita and population. GDP per capita growth is derived from three sources. The first is the ENV-Growth model SSP2 ("middle of the road") by the Organization for Economic Co-operation and Development (OECD[4]; the second is the International Institute for Applied Systems Analysis (IIASA) model SSP2[5] and the last one is based on recent history (1991-2010) trend line linear extrapolation, hereafter called LINE. The projections from OECD and IIASA are in constant 2005$ and can be converted to match the World Bank unit in constant 2010$ with an inflation rate of 1.12.[6] Population is based on the UN projection under the medium-fertility variant

scenario.[7] All the future projections are divided by their own 2010 estimates to obtain the growth percentages.

Although wood consumption has a generally positive relationship with GDP per capita, some high-income countries, such as Australia, Canada, Japan, and the United States, saw decreases in their historical per capita consumption of sawn wood, wood-based panels, and paper and paperboard consumption as their GDP per capita grew beyond certain levels. We therefore separated the countries into developed and developing countries to avoid overestimating future wood consumption in high-income countries. We used a threshold of USD $40,000 for sawnwood and wood-based panels, and a threshold of $12,000 for paper, paperboard, and wood fuel. We chose $12,000 for paper and paperboard and wood fuel because the threshold for high-income countries is $12,615 by the UN definition. For sawn wood and wood-based panels, we found that $40,000 is a better threshold for model fitting to group the responses of wood consumption to GDP and population.

In each FE regression model, we have dependent variable wood consumption and multiple predictor variables. We used two types of formulas (Equations 4-5): one only depending on the GDP and population and the other one including the effect of development and years after 2000 as a proxy for technological and policy changes after that date. We selected the year 2000 because the transitions of wood consumption growth in many countries occur around 2000, when the internet usage boom started and modified paper needs. The wood consumption is log transformed (natural), and two predictor variables, GDP per capita and population, are log transformed.

$$\log(W_{it}) = \alpha_i + \beta_1 \log(G_{it}) + \beta_2 \log(P_{it}) \tag{4}$$

$$\log\,(W_{it}) = \alpha_i + \beta_1 \log(G_{it}) + \beta_2 \log(P_{it}) + \beta_3 Y_t^n + \beta_4 Y_t^s \tag{5}$$

*W* is the wood consumption per capita of each product type (tons per capita), and *G* is GDP per capita (US$ per capita). The index *i* refers to the country, and *t* refers to a data point in time, meaning year = 1961, …, 2020 in this study. The expression $\alpha_i$, *i* = *1, ..., n*, can be understood as the unobserved time-invariant heterogeneities across the countries *i=1, ..., n*. These individual specific intercepts are considered the fixed effects of countries. $Y_t^n$ is the number of years since 1961, and $Y_t^s$ is the number of years after a shifting technology takes place. Holding the variables related to time trends constant, the ratio of wood consumption between two countries $(W_1/W_2)$ equals the ratio of GDP per capita $(G_1/G_2)$ to the power of $\beta_1$, multiplying the ratio of population $(P_1/P_2)$ to the power of $\beta_2$. It tells us that if the ratios of GDP per capita and population remain the same, the ratio of wood consumption stays the same too. Otherwise, the combined effects of GDP per capita and population on wood consumption are no longer linear. The variables related to years are not log transformed because they have zero values. It means that, for one year increase in the number of years since 1961, it is expected to see a value of (exp($\beta_3$) – 1) increase in wood consumption.

In summary, we established 12 relationships ("models") based on three different types of wood products, two different trend lines in developed and developing countries, and two different regression formulas, one incorporating year as an independent variable and one without (Supplementary Table 2). The FE model parameters $\beta_1$, $\beta_2$, $\beta_3$, and $\beta_4$ and goodness of fit are estimated by the ordinary least squares regression model with *n - 1* dummy regressors using the R packages "lm" and "lfe." We obtained an output of a global slope for each model and individual $\alpha_i$ for each country *i*. Supplementary Table 3 provides the coefficients ($\beta_1$-$\beta_4$) for the independent variables and their two-sided t-test statistics. All the models have high $R^2$ full (> 0.88) and significant P values (< 0.05) and have a residual standard error (RSE) between 0.32 and 0.84 (Supplementary Table 2). $R^2$ full is the typical $R^2$ between all pairs of FE-predicted values and original values. For the FE model, another goodness of fit $R^2$ projection is also considered, which means how much of the variation in the dependent variable for each country is captured by the model. $R^2$ projection is expected to be small. Paper and paperboard per capita has the highest $R^2$ projection, and wood fuel per capita has the lowest $R^2$ projection, which means the time trend cannot explain the variations of wood fuel very well. The FE models have good predicting power in developed countries for sawn wood, wood-based panels, and paper and paperboard and in developing countries for wood fuel (RSE < 0.4).

We interpreted these as indicative relationships. In theory, the quantity of wood use could drive GDP growth rather than the other way around, but because wood consumption is a small part of overall GDP growth, that is unlikely. And even if both wood use and per capita income were driven by a third, unknown factor related to both, per capita income growth could still be a good predictor of future wood use.

Based on the coefficients for the models with the time effect, we can derive the wood consumption in the 2010 reference year and the 2050 projected year as follows:

$$\log(W_{i,t=2010}) = \alpha_i + \beta_1 \log(G_{i,t=2010}) + \beta_2 \log(P_{i,t=2010}) + \beta_3(2010 - 1961) + \beta_4(2010 - 2000) \quad (6)$$

$$\log(W_{i,t=2050}) = \alpha_i + \beta_1 \log(G_{i,t=2050}) + \beta_2 \log(P_{i,t=2050}) + \beta_3(2050 - 1961) + \beta_4(2050 - 2000) \quad (7)$$

Subtracting wood consumption in 2010 from 2050 leads

$$\log\left(\frac{W_{i,t=2050}}{W_{i,t=2010}}\right) = \beta_1 \log\left(\frac{G_{i,t=2050}}{G_{i,t=2010}}\right) + \beta_2 \log\left(\frac{P_{i,t=2050}}{P_{i,t=2010}}\right) + (\beta_3 + \beta_4)(2050 - 2010) \quad (8)$$

$W_{i,t=2010}$ is the 2010 reference wood consumption, which is the 2006–2014 average of the annual wood consumption. $\frac{G_{i,t=2050}}{G_{i,t=2010}}$ is the ratio of GDP per capita between 2050 and 2010, and $\frac{P_{i,t=2050}}{P_{i,t=2010}}$ is the ratio of population between 2050 and 2010 from the United Nations. The 2050 wood consumption in each country is derived from the above formula for three GDP per capita projection models (OECD, IIASA, and LINE) and for two regions (developed and developing). Similarly, the 2050 wood consumption for the models excluding time effect can be derived as this simplified formula as Equation 9:

$$\log\left(\frac{W_{i,t=2050}}{W_{i,t=2010}}\right) = \beta_1 \log\left(\frac{G_{i,t=2050}}{G_{i,t=2010}}\right) + \beta_2 \log\left(\frac{P_{i,t=2050}}{P_{i,t=2010}}\right) = \log\left(\left(\frac{G_{i,t=2050}}{G_{i,t=2010}}\right)^{\beta_1}\left(\frac{P_{i,t=2050}}{P_{i,t=2010}}\right)^{\beta_2}\right) \quad (9)$$

GDP per capita from the complex model projections are dramatically high in developing countries, and the GDP per capita from the simple linear model may be too low in developed countries. To avoid the unrealistic overestimation of future wood consumption, we first applied a cap to the developing countries' wood consumption per capita using the 75th percentile of the developed countries' wood consumption per capita in 2050. After capping the developing countries, we further filtered the unlikely high wood consumption per capita that has more than a 10-fold increase between 2010 and 2050. Then we obtained the intermediate prediction by applying equal weights to the results based on complex models (OECD/IIASA) and recent linear extrapolation (LINE). In other words, the weights for OECD, IIASA, and LINE are 0.25, 0.25, and 0.5, respectively. The uncertainty of choosing the projection of GDP per capita is discussed in Section 5.3.

Considering the combination of matching FAOSTAT recent trends and higher $R^2$, for sawn wood and wood-based panels, we selected the regression formula with the time effect for developed regions and without the time effect for developing regions; for paper and paperboard, we selected the regression formula with the time effect for both regions. For wood fuel, we calculated the average between the two formulas in developing countries. In developed countries, we used the formula excluding time effect for wood fuel because the recent increasing trend in wood fuel is related to short-term policy and should not be built into the model for long-term projection. Finally, we obtained the average national growth percentages from 2010 to 2050 for the consumption of three wood products.

**Estimating future harvest levels**

We applied the growth percentages of sawn wood and wood-based panels, paper and paperboard, and wood fuel to consumption of LLPs-M (main), SLPs (wood pulp), and VSLPs-WFL. We keep the LLPs-O (other) unchanged between 2010 and 2050 because there are no available trade statistics for other industrial roundwood, and we cannot assume LLPs-O grow at the same rate as LLPs-M. Note that this can underestimate the real wood demand. We keep wood pulp growing at the similar rate as paper and paperboard under the assumption that the ratio of wood pulp to paper remains unchanged between 2010 and 2050.

The results of this FE model are the consumption of each wood product category in 2050. However, the inputs for CHARM are the amount of wood production. To predict the production in 2050 for CHARM inputs, we assume the trade balances in 2050 are the same as the 2010 reference. The effect of different future trade patterns is discussed in Section 5.4. We first split the countries in 2010 into net importers (net imports < 0) and net exporters (net exports > 0). For net importers, we calculated the import percentages (net imports/consumption) and apply these percentages to the 2050 consumption to get 2050 net exports. For example, if a country imports

20% of its wood in 2010, the model assumes it will do so in 2050. After that, we calculated the 2050 world total net exports (= sum of world total net imports). For net exporters, we calculated the 2010 export shares of global exports (net exports/world total net exports) for each country. We adjusted the 2050 net exports of these countries in response to match the 2050 world total net exports in proportion to their share of global exports. Finally, we derived the 2050 production using 2050 consumption and 2050 net exports for both net importers and net exporters.

For other industrial roundwood, LLP-O and VSLP-IND-O 2050 production remains the same as 2010 production. To estimate industrial wood waste (VSLP-IND-M) production in 2050, we calculated the ratio of VSLP-IND-M to IND-M. Then we calculated the difference of IND-M 2050 and IND-M 2010, and then applied the ratio to this difference and got the additional waste (VSLP-IND-M 2050 – VSLP-IND-M 2010). At the end, we obtained the total VSLP-IND 2050 production by adding up VSLP-IND-M 2050 and VSLP-IND-O 2050.

Since our model requires roundwood harvest, we expanded the wood demand quantities into roundwood equivalents by considering the bark (overbark to underbark ratio = 1.15, 15% more mass). The model requires not only data on absolute roundwood harvest but on product categories. Not all countries have data for all the different product categories, and a few countries have product category data that is clearly unreliable. For this reason, we restricted our analysis to those 30 countries with the best data that account for 80% of global wood harvest. We sorted the country-level results by 2010 production from greatest to least and use the top 80% countries across the three product categories. Extended Data Table 3 lists the consumption of different wood products by the 30 countries in 2010 and as projected in 2050.

**Comparison with other wood demand projections**

Our projections are mostly within the range of growth rates projected by other published studies (Supplementary Table 4). For example, Szabó et al. (2009)[8] projected a 243% increase in use of paper and paperboard between 2000 and 2030 in Asia and a 200% increase in South America. Over a 40-year period from 2010 to 2050, we project a 180% increase in East Asia and 249% increase in Latin America. FAO (2009)[9] projected that global consumption of sawn wood and wood-based panels would increase by 41% and 116%, respectively, from 2005 to 2030, whereas our projections are for an 84% increase in sawn wood and wood-based panels from 2010 to 2050, which combines both items and covers 40 years rather than 25 years[9].

Buongiorno (2015)[10] projects that the world is likely to demand about 50% more industrial roundwood by 2050 relative to 2010, lower than our estimated 88% increase. Compared to Buongiorno (2015), we projected similar changes for LLPs in Europe and North America, but much higher growth rates in other regions. One explanation may be that we used more recent, higher projections of GDP per capita and population growth rates (rising to 9.7 billion rather than 9.3 billion in Buongiorno [2015]). We also used a fuller length of historical data (1961–2020). Buongiorno (2015) used the shorter period of 1992–2012, which ended in years of recession with depressed wood use. Compared to that study, we also projected a larger increase in paper consumption in Africa, Asia, and Latin America and a similar increase in Europe and North America.

Our projection of 22% in direct use of wood for fuel compares with only 1% in Buongiorno (2015). Wood fuel use has the least consistent relationship with growth in population and GDP per capita. China, for example, mostly shifted from wood fuel to fossil fuels despite a relatively low per capita income, but low-income African countries have continued to rely primarily on wood fuel. Because of this variation, and because future wood fuel use will depend greatly on government energy policies, we consider our wood fuel projection (and any wood fuel projection) to be the least reliable of overall wood consumption projections.

Although our model has reasonably good statistical fits, wood consumption levels depend on many unknown variables, and future wood consumption is likely to depend on factors that cannot be predicted with present information. One unknown is the effect of changing technologies. For example, Hurmekoski and Hetemäki (2013)[11] argued that the structural change driven by digital information technology around 2000 has had large downward impacts on paper demand. Studies using data before 2010 cannot fully account for these trends and therefore could not project the effects of changing technology. On the other hand, more than 50% of paper products are used for packaging, and the global rise of internet shopping could fuel increases in paper used for packaging[12]. Another uncertainty is possible constraints on supply. In the Buongiorno (2015)[10] model, projected wood price increases depress growth in future wood consumption. These price increases may occur, but to our knowledge, there is no good econometric analysis of the long-term supply and demand elasticities with which to project future wood prices.

## 2. Wood harvest and land use

The land area requirements for the model are calculated at the national and global levels. Demand for different types of wood products per year is provided as an input, converted into roundwood equivalents, and then used to estimate wood harvest. Wood is supplied from one of two sources, plantation forests and secondary forests, each with its own efficiencies of wood harvested. Wood supply from plantation forests is used first, with remaining forest supplied by secondary forests.

To estimate wood supplied by secondary forests, the forest types in each country are characterized by their aboveground growth rates, areas, and some other characteristics, and a composite national-level forest type is created by the weighted average of the secondary forests. (The result is mathematically equivalent to allocating wood harvests to each separate forest type based on its percentage area.) Wood supply from each hectare is provided by this national-average forest based on the percentage of aboveground wood harvested that makes it into a product pool while the remainder is left as slash.

Natural forest carbon stocks at time of harvest can be varied. For our present scenarios, we assume that only secondary forests will be harvested, and they are harvested at least after 40

years or 20 years growth after reaching the national average aboveground biomass for secondary forests.

For plantation forests, initial wood supply in 2010 is based on the area of planted forest estimated by the FAO divided by the estimated average rotation length. For example, if the rotation length is 10 years, then a 10th of the plantation forest is estimated to be available in 2010 and in subsequent years. Plantation slash rates are established separately. Plantation forests can also be thinned, with some of the wood harvested in this way available for SLPs or VSLPs.

Different scenarios allow plantation areas to evolve over time according to different rules. For example, in one scenario, new plantations come from agricultural land. In another, secondary forests are converted to plantation forests as secondary forests are harvested. Because plantation forests need to grow before they can supply wood, the supply from plantation forests can be constrained. The model estimates the potential supply of wood from plantation forests each year between 2010 and 2050 and allocates the remainder of the supply to secondary forests. Model results for each country include the total area of plantation forests that will be established in 2050 and the total hectares of harvests of secondary forests that must occur between 2010 and 2050 to meet wood product demands.

Wood demand and supply is estimated for the world's top 30 wood-producing countries because of the higher quality of data available for those countries. Together, these countries made up around 80% of the world's wood production in 2010. By dividing the wood demand by 0.8, we then adjusted from these 30 countries to estimate 100% of global wood harvest. Implicitly we are assuming that the 20% of wood harvest uses that we cannot further analyze match the characteristics of the 80% we can. Supply is met from within the country based on its share of demand produced internally, and imports are met proportionately by exporting countries. We also divided the areas by 0.8 to generate global estimates, which assumes that the remaining 20% would be met with a harvest efficiency equal to the average of the other 80%.

Below is a mathematical description of land area calculation process. For each scenario, we calculate the total number of hectares required for harvesting every year from 2010 to 2050. To do this, we first calculate the total amount of each product required every year in each product pool (LLP, SLP, VSLP) using Equation 10.

For each product pool $j$ in year $i$,

$$W_{i,j} = W_{j,i-1} + I \tag{10}$$

where $i$ is the year in the range of 2011–50, $W$ is the tons of dry matter of a wood product type $j$ produced in year $i$ (the dry matter in product pool $j$ in the year 2010 is calculated based on the ratio of LLP:SLP:VSLP in the 2010 baseline), and $I$ is the annual proportion of increased demand calculated as

$$I = \frac{W_{2050} - W_{2010}}{2050 - 2010} \tag{11}$$

We then convert the total tons of dry matter in all product pools into tons of carbon based on the assumption that dry matter is 50% carbon.

We assume that there is a maximum number of plantation hectares that may be harvested such that all hectares are harvested over the course of a single rotation period. For example, if a country has an average rotation period of 10 years, every hectare may be harvested four times over 40 years, and no more than 10% of managed forests may be harvested each year.

For a small number of countries where there is a large area of plantation forest, and supply for a given year is less than the maximum production capacity from plantation hectares, the number of hectares harvested is scaled down accordingly to eliminate any surplus. For example, if a country with a rotation period of 10 years can harvest up to 100 ha every year with a capacity of 1,000 tC in products per year, but the supply needed in a certain year is only 900 tC, then the model would only simulate the harvest of 90 ha. For most countries, plantation forests are not sufficient to meet demand, and the remainder is assumed to come from secondary forests. For example, if the supply needed is 1,100 tC, then 100 ha of plantation would be harvested, and the rest of the wood would come from secondary forests.

After calculating the amount of wood supplied from plantation forests in a given year, we determine the number of secondary forest hectares required if all supply is not met from the first or subsequent harvest of plantations:

$$a_{secondary} = \frac{W_r}{P} \tag{12}$$

where $P$ is the wood product yield, or the amount of aboveground biomass that makes it into a product pool in units of tons of carbon per hectare of secondary forest, and $W_r$ is the remaining amount of carbon required that is not supplied by plantation forests.

The sum of the area required every year from 2010 to 2050 is the total area harvested in the period of this study.

## 3. Biophysical Model Inputs

Our model requires separate biophysical inputs for secondary forests and plantations. For both forest types, we create an “average forest” for each country, which includes the growth rate as an average across all ecozones weighted by area. Harris et al. (2021)[13] combined a variety of sources to develop a dataset on forest growth rates by area. The resulting data set provides many growth rate parameters, some of which are integral to our analysis. For any given country and ecozone (tropical, temperate, etc.), the model provides the forest type (primary, young secondary, old secondary, or plantation), area, aboveground carbon stock across the entire area, aboveground carbon density per hectare, and annual growth rate per hectare.

## 3.1 Secondary forest growth function and parameters

For secondary forest growth rates, Harris et al. (2021)[13] provides two growth rates: less than 20 years of age (GR1) and greater than 20 years of age (GR2). We used the estimates and adjusted them based on the following rules. If the ratio of GR2 to GR1 is larger than 85%, or even if GR2 is larger than GR1 (> 100%), we utilized Bernal et al. (2018)'s GR2/GR1 ratio[14] and calculated the average GR2/GR1 between the two data sets (see summary in Supplementary Table 5).

We used the Monod function to simulate the higher growth rates in the younger forests and lower growth rates in the older forests[15,16]. Because we are discounting growth by time, higher growth rates for younger forests (versus older forests) matter to our calculations. For growth rates beyond 20 years, the data set developed by Harris et al. (2021).[13] includes not only forests that are just modestly over that threshold but also older secondary forests with slower growth rates because this limited categorization served the purposes of the study. The secondary forest grows at a Monod function of forest age[15]:

$$C(Age) = \frac{AGB_{max} * Age}{Age + Age_{50\%}} \tag{13}$$

The parameters maximum AGB ($AGB_{max}$) and the number of years to reach half saturation AGB ($Age_{50\%}$) are solved using the GR1 and GR2 from Harris et al. (2021)[13] and Bernal et al. (2018)[14]. The initial carbon stock for secondary forests being harvested depends on the age of harvest $Age_{harvest}$, and is set at additional 20 years growth after the average aboveground carbon stock from the Harris et al. (2021)[13] data set with a minimum of 40 years.

## 3.2 Existing plantation forest growth rates

For an existing plantation forest, we first used the growth rates from the Spatial Database of Planted Trees (SDPT Version 1.0)[17] in Harris et al. (2021). By the definitions of SDPT, "wood fiber" type is the planted forests for timber products, and "Not applicable" is non-planted forests. We weighted average "wood fiber" type by area in each country and used the average growth rate for young and older forests.

From the SDPT, 16 of the 30 countries have available plantation growth rates. For the remaining countries (mostly in EU, boreal region, and Africa), the missing plantation growth rates are largely due to the lack of planted forest area data. There are substantial data gaps[17] between satellite data and FAO Global Forest Resources Assessment (FRA) in boreal region like Canada and Russia and sub-Saharan Africa. For Canada and Russia, although FAO lists millions of hectares of planted forests, there is little intensively managed plantation.[17] In these countries as in several countries in Europe where plantation rotation lengths are more than 40 years, it is not possible to separate the growth rates of planted forests listed by FAO from the growth rates of secondary forests overall. We therefore use the average forest secondary growth rate for plantations in these countries. For sub-Saharan Africa, seven countries including Ethiopia, Ghana, Kenya, Nigeria, South Africa, Uganda, and Tanzania, SDPT has either no or little spatial coverage, while the FRA reports much larger plantation area based on expert assessment. We decided to use the young secondary growth rate GR1 for these countries as their rotation lengths

are shorter than 40 years. (The areas of planted forests in these countries according to the FAO data are also too modest to have significant effects on global results.)

For countries with large plantation areas that could more meaningfully impact overall results, and for which there is conflicting evidence, we consulted additional sources. Particularly, we sought additional information on plantation growth rates for Brazil, China, Mexico, Indonesia, and the United States. In the first four countries, data on growth rates is based primarily on harvested wood delivered to processing facilities. To convert that to forest, above-ground carbon sequestration rates, we used

*2003 IPCC Good Practice Guidance for Land Use, Land-Use Change and Forestry* (GPG-LULUCF) Equation 3.2.5[18]:

$$GR_c = MAI \cdot WBD \cdot BEF \cdot CW \quad (14)$$

where the growth rate in carbon $GR_c$ is annual aboveground carbon density (tC $ha^{-1}$ $yr^{-1}$), *MAI* is the mean annual increment ($m^3$ $ha^{-1}$ $yr^{-1}$) in merchantable wood volume (excluding branches and leaves), *WBD* is the wood basic density (t dry matter $m^{-3}$), *BEF* is the biomass expansion factor for conversion of annual net increment (including bark) to aboveground biomass, and *CC* is the carbon fraction of dry wood (= 0.5 t C per t dry matter). We tried our best to find regional average mean annual increment data from national report and literature, as well as measurements over large-scale. We avoided the growth number obtained in scientific productivity experiments, as they are almost always higher than the actual national average for production and not meaningful for our purpose. Where more localized information was not available, we used the look up table from IPCC GPG-LULUCF Annex Table 3A.1.10[18] for BEF values.

**Brazil**

IBA, the association of the Brazilian Tree Industry (IBA), provides annual reports with information on planted forest area by type and consumption of wood by facilities that harvest this wood. Our estimate of growth rates per hectare uses 2012 information on planted forest area, separately provided for eucalyptus, pine and other, and 2016 information on quantities of wood consumed. (Planted area in 2012 is provided in the 2014 report[19], and quantities consumed is provided in the 2022 report[20].) We used this lag to recognize that because Brazil's area of planted forest is growing, some of the planted forests in 2012 would be newly planted and would not be generating harvests in 2012. Because the wood consumed is only the wood harvested, we also used a BEF to estimate total above ground carbon. In Brazil, the great majority of plantation forest wood is used for pulp or charcoal. That allows highly efficient uses of above-ground carbon reported at 88% by Greenwood Resources, a major owner and operator of Brazilian forest plantations, which converts to a BEF of 1.14. We applied this BEF both to eucalyptus and pine and used a higher BEF of 1.35 for other. (The IBA reports also indicate low levels of slash.) The final calculation results in an estimate of 8.22 tC $ha^{-1}$ $yr^{-1}$ above-ground forest gains.

**China**

We collected statistics from the literature based on the Chinese National Forest Inventories. We gathered the annual volume increment ($m^3$ $ha^{-1}$ $yr^{-1}$) for top 10 plantation tree species within the

young and middle-aged range[21]. We found corresponding plantation area (ha; [22]) and wood basic density (t $m^{-3}$; see [23]) along with IPCC BEFs. We generated a weighted average growth rate of 1.27 tC $ha^{-1}$ $yr^{-1}$ for existing plantation. This growth rate is smaller than the young secondary forest growth rate because the widely planted forests are mostly in the arid climate and under relatively poor nutrition/management at large scale.

**Indonesia**

We used the mean annual volume increment in Harwood and Nambiar (2014)[24] for the two main species Acacia and Eucalyptus in Indonesia. Although Acacia has been the dominant species historically, various papers indicate that there is a transition from Acacia to Eucalyptus because of the high mortality rates in Acacia. We assume a 50:50 area share for the two species going forward. We used the wood basic density[25,26] and BEF from Miyakuni et al. (2004)[24] and IPCC[18] to obtain an above-ground plantation growth rate of 7.21 tC $ha^{-1}$ $yr^{-1}$.

**Mexico**

We used the annual volume increment and area for six major plantation species from the report of the Mexican National Forestry Commission.[27] We supplemented the data with the IPCC BEFs and wood basic density from Harris et al. (2021) and aggregated the growth rate to the national average plantation growth rate at 3.60 tC $ha^{-1}$ $yr^{-1}$.

**United States**

For U.S. plantation growth rates, Harris et al. (2021) used estimates of growth rates from U.S. Forest Service inventory data of different forest types indicated to have had artificial regeneration and without indications of disturbance. These data sources resulted in an estimate of 3.85 tC $ha^{-1}$ $yr^{-1}$ of above-ground carbon gains as a weighted average of different plantation types. However, these growth rates were substantially higher than key plantation types estimated from the same data source for all loblolly and other plantation types in the southeastern United States in the 2017 Forest Resources Assessment[28]. They were also substantially higher than the carbon accumulation rates of high productivity stands of the four most widespread plantation types in a U.S. Forest Service Publication (Hoover et al. 2021).[29] One explanation may be the complete exclusion of disturbed used for Harris et al. For our purposes, we need to exclude areas with signs of harvest but include areas of natural disturbance. For the three most prevalent planted forest types, which comprise 82% of the total U.S. planted forest area as estimated by Harris et al. (2021), we found a 42% difference between estimates from that analysis and those for high productivity sites in Hoover et al. (2021). We averaged the two results and accordingly reduced the Harris et al. (2021) estimated growth rate for all plantations by 21%, yielding an average plantation growth rate of 3.05 tC $ha^{-1}$ $yr^{-1}$ in above-ground carbon.

**Plantation areas**

The SDPT V1 used in Harris et al. (2021) has no reported hectares of plantation forest in some countries or has large discrepancies with FRA data. To overcome this issue and maintain consistency, we used the area of managed forest provided by FRA for the 30 countries.

**Summary of forest growth rates**

Supplementary Table 5 lists the national weighted average growth rates of secondary and existing plantation forests, average secondary carbon stock, and plantation areas for the 30 countries. Existing plantation growth rates could be much smaller than secondary forest in some cases (e.g., China, India) because plantations are established on generally drier lands than remaining secondary forests. For our Scenario 2 (converting secondary forest to plantation forest), this assumption of lower growth rates would not make sense because the plantation is established in the same areas as the secondary forest. In these countries, we therefore used the young secondary forest growth rates as the converted plantation growth rates instead of the existing (lower) plantation growth rates.

## 3.3 Harvest parameters

**Rotation period**

A key parameter relevant to management is the rotation period for both the harvests and the thinnings. At present, we apply parameters for thinnings to some stand-level analyses but because of limited information on the extent of thinnings do not apply thinnings to the global scenarios; however, the effects of thinning are implicitly incorporated into estimated growth rates and harvest volumes.

The rotation period is a highly variable parameter that depends on the specific management regime for a given plantation type. For a stand-level scenario, users can input a specific rotation period. However, we consulted the literature to find the best estimate for each country for our global analysis. Supplementary Table 6 summarizes the sources used to determine the rotation periods that we used for our global scenarios. It should be noted that this information was not readily available for some countries. In these situations, we made educated guesses based on the plantation growth rates and the known rotation periods of other countries.

**Slash rate**

The model also requires information on management decisions and harvest efficiency. The model requires the proportion of wood from a harvest or thinning that makes it into each product pool, how much AGB is left as slash after a harvest, and for stand but not global analysis, the proportion of AGB that is removed during the thinning. When we estimated plantation growth rates, we used the biomass expansion factor (BEF) to estimate the branches and leaves, which is the slash proportion (BEF - 1 = slash). In order to be consistent with our plantation growth rate, slash rates should match this equation: (BEF – 1)/BEF. Our BEFs are mostly based on IPCC as discussed above and supplemented by literature review.

For the secondary forest slash rate, the model uses a default value of 20% for the VSLP products. For LLP and SLP, the model uses a 30% for Asian countries and a 25% for EU and North American countries with a higher harvest efficiency.  In tropical countries, where selective harvest predominates, the slash rate is far higher.[30] At present, we apply country-specific secondary forest slash rates (Supplementary Table 7) to 16 tropical forests based on references [30] and [31].

### 3.4 Decay parameters

We applied decay rates for each carbon pool post-harvest according to Table 12.2 of the *2006 IPCC Guidelines for National Greenhouse Gas Inventories*[32] and other literature, described in Supplementary Table 8. However, these values can be modified for more specific scenarios. Annual emissions are calculated by tracking the decay that occurs in each pool from one year to the next, including methane due to landfilled LLPs, as described in the Methods.

#### Storage in construction

The quantity of carbon stored in wood products depends in part on the share of LLP used for construction and the persistence of this wood. For both, we used estimates Zhang et al. (2020)[33]. This paper developed a new method for estimating the percentage of LLPs that are used in construction by mapping FAOSTAT production data to the Eora Global Supply Chain Database's consumption data. They estimated the quantity of wood used in construction for the top 10 hardwood-product-producing countries (all of which are included in our analysis). For all other countries that produce hardwood products, they provided a single ratio. The ratios for the top 10 countries and the remainder are presented in Supplementary Table 9.

Zhang et al. (2020)[33] also provide half-lives derived from a meta-analysis for several different countries, many of which are relevant to our model. Where this information is not available, Zhang et al. defer to the IPCC assumption that LLPs in construction have a half-life of 40 years, whereas all other LLPs have a half-life of 23 years. Supplementary Table 10 shows the half-lives for construction material and other LLPs for each country.

CHARM calculates the benefits due to avoided emissions from concrete and steel in construction by estimating the percentage of LLPs in a country that are used for construction and uses estimates of the quantity of construction material that displaces concrete and steel. This value is uncertain because the quantity of wood that replaces a given amount of concrete and/or steel varies widely by region and building type.[34] Chen et al. (2018)[35], however, estimates that 64% of LLPs used in construction displace concrete and steel in Canada.

We used these half-lives to calculate a weighted average half-life based on the percent of LLPs in construction in Supplementary Table 10. The calculation is as follows: (% LLP in construction × half-life for LLP in construction) + ([1 – % LLP in construction] × half-life for other LLP). The weighted average half-lives for LLPs are between 12 and 47 for the 30 countries.

## 4. Model Scenarios

### 4.1 Global wood supply scenarios

For our global analysis, we analyzed seven different wood supply scenarios. For example, Scenarios 1 and 2 explore the effects of changes in timber production and the difference between allowing a natural forest to regenerate after harvesting rather than converting it to a plantation. For each scenario, we calculated the carbon impacts and land-use requirement with two supply levels. In the first supply level, timber supply remains constant at 2010 levels, and "BAU" means that timber supply changes according to a business-as-usual projection.

- **Scenario 1 (secondary forest harvest and regrowth)** assumes that the existing plantations are supplying wood at our best estimate of their present growth rates. Additional wood demand is met by the harvest of wood from middle-aged secondary forests (stands aged 20–80 years) and the forests are allowed to regrow for 40 years. This scenario also assumes that all wood is supplied by at least small clear-cuts, and it measures the area of such clear-cuts.

- **Scenario 2 (secondary forest harvest and conversion)** assumes that the existing plantations are supplying wood at present growth rates and that after secondary forest areas are harvested as Scenario 1, they are reestablished as plantations (assume at productive locations with at least the present growth rates of secondary forests) to maximize the amount of future wood supplied by plantations. Scenario 2 is a bounding exercise for high levels of intensification. Plantations have substantially higher output of wood per hectare per year and are typically harvested more efficiently than natural forests, which means that more of the wood felled is utilized for wood instead of being left as deadwood in the forest. This scenario is designed to analyze the effects of a high level of intensification in forest management.

  Although we assume that the same lands are replanted as plantations, something similar to this scenario could also occur if natural forests continue to be cleared in some areas while plantations are regrown in others. In China, for example, as discussed above, the large-scale conversion of less productive agriculture lands to plantations is associated with a heavier reliance on imported foods associated with a large quantity of offsetting deforestation[36]. On a global basis, growth of plantations on abandoned agricultural land can therefore indirectly achieve a conversion of natural forests to plantations.

- **Scenario 3 (secondary forest mixed harvest)** is similar to Scenario 1 except that 50% of wood demand is provided by middle-aged secondary forests (20–80 years) and 50% is provided by mature secondary forests (80–140 years). Both secondary forests are harvested at the same slash rates. This is the same as Scenario 1, except that the wood supply from the secondary forest is partially sourcing from mature forests as well.

- **Scenario 4 (new tropical plantations)** assumes that 2 Mha per year of tropical agricultural lands become available for establishing highly productive plantations in the tropics and are harvested evenly between 2020 and 2050 (a total of 68 Mha are established given the average tropical rotation period at 7 years). All new plantations occur in existing agricultural lands in the tropics and neotropics, where yields are higher. The secondary forests are harvested less due to the wood supply from the new tropical plantations. This scenario assumes that these lands have been spared from agriculture, so the carbon costs of using these lands for plantations is the loss of carbon sequestration that would otherwise occur in regrowing secondary forests.

- **Scenario 5 (higher plantation productivity)** is identical to Scenario 1 but assumes that existing plantation forest growth rates increase by 25% between 2010 and 2050.

- **Scenario 6 (higher harvest efficiency)** is identical to Scenario 1 but assumes that existing tropical secondary forest harvest efficiency increases so that the slash rate reduces to the level of best practices as described by Ellis et al. (2019).

- **Scenario 7 (Reduced less wood fuel demand)** is a variant of Scenario 1 in which wood fuel demand in 2050 reduces by half compared to the demand under the BAU projection in Scenario 1.

### 4.2 Harvest and non-harvest growth rates

In the different scenarios, secondary forests are harvested and allowed to regrow, secondary forests are converted to plantations, existing plantations are harvested and allowed to regrow, and agricultural land is converted to plantations. Supplementary Table 11 describes the growth rates used for the harvest and non-harvest conditions for each of these forest harvest types.

## 5. Sensitivity Analyses

### 5.1 Growth rates

We examine the impacts of higher or lower absolute and relative forest growth rates (Supplementary Table 12). Altering absolute and relative growth rates is implicitly also a way of altering when the forests will be harvested. Our analysis shows that increasing absolute forest growth rates by 25% increases emissions by 13% and altering them down by 25% lowers the emissions by 12%. The 25% change in growth rates result in a range of 3.60 to 4.64 $GtCO_2e\ yr^{-1}$ emissions, and 767-961 Mha global land requirements.

We also evaluated the potential effects from altering the ratio of growth rates for young and older forests. GR1/GR2 is the ratio of young forest growth rate to older forest growth rate. A 25% increase in this ratio, meaning higher young forest growth rate than older forest growth rate, decreases by 10%. We also explore a 50% increase in this ratio in which, for example, the average growth rates for secondary forests in the United States in the first 20 years is ~4x faster than the growth rate over the succeeding 40 years, which is vastly greater than the differences reported in the literature.[37] Even in this scenario, the annual emissions are reduced by 18% and are therefore still projected at 3.36 $GtCO_2e\ yr^{-1}$.

### 5.2 Root-to-shoot ratio

Our current estimate of belowground (root) to aboveground (shoot) biomass ratio is based on the power function set forth in reference[38]:

$$BGB = 0.489AGB^{0.89} \tag{15}$$

Altering global root to shoot ratios of secondary forests 25% up or down alters emissions by 6% up or down (Supplementary Table 13). The 25% change in growth rates result in a range of 3.88 to 4.33 $GtCO_2e\ yr^{-1}$ emissions. For a forest holding 70 $tC\ ha^{-1}$ aboveground carbon, the global average root to shoot ratios now vary from 0.21 to 0.35. The emissions go up when root to shoot

ratio increases because more roots are disturbed and plus the fact that roots decay faster than some large pools like LLP products.

## 5.3 Economic growth model

Our current projection of wood demand is based on the weighted average of the projection using three economic growth models. To present the uncertainties of choosing different economic growth model, we run the simulations using three models separately. (To avoid the unrealistic overestimation of future wood consumption, we first applied a cap to the developing countries' wood consumption per capita using the 75th percentile of the developed countries' wood consumption per capita in 2050.) The OECD and IIASA economic growth models predict a growth of 110% to 114% in global GDP per capita, and the LINE model (a pure trend line model) predicts a growth of 37%.

Supplementary Table 14 shows that the projections using OECD and IIASA SSP2 predict 18-20% higher BAU wood demand than current projection, while using LINE reduces 16% of BAU demand between 2010-2050. Using higher projected economic growth rate such as OECD increases BAU wood demand by 18% and results in 14% higher projected future emissions and 25% more land use. Applying linear economic growth rate reduces BAU wood demand by 16% and results in 12% lower projected future emissions and 22% less land use. Different increases in cumulative wood demand do not result in exactly proportionate increases in emissions because they have different effects on each wood product category and have different effects on countries, which have different wood use efficiencies.

## 5.4 Trade patterns

Our current 2050 projected wood production assumes existing trade patterns remain without better knowledge. We examined the potential impacts of varying future trade patterns on our carbon and land estimates. We adjusted the share of wood exports from tropical countries from the top exporter countries (15-30% of the world industrial roundwood production) by 50% up or down. This only alters the emissions in our Scenario 1 by less than 1% up or down (Supplementary Table 15).

## 5.5. Overall sensitivity results

The above additional sensitivity analyses are summarized in Extended Data Figure 4. It shows that under a broad range of possible parameters, the annualized global emissions using our timing approach remain in the 3-5 $GtCO_2e\ yr^{-1}$. In an extreme scenario, these emissions decline "only" to 3.36 $GtCO_2e\ yr^{-1}$.

# B. Supplementary Tables

## Supplementary Table 1. FAOSTAT Items and Elements

| **Country *N* in year *T*** | **Industrial Roundwood (IND)** | **Sawn Wood (SNW)** | **Wood-Based Panels (WBP)** | **Wood Pulp (WPL)** | **Other Industrial Roundwood (IND-O)** | **Wood Fuel (WFL)** |
|---|---|---|---|---|---|---|
| **Production** | Y | Y | Y | Y | Y | C |
| **Net exports** | Y | Y | Y | Y | - | - |
| **Consumption** | C | C | C | C | C | - |

*Notes:* The data directly from FAOSTAT are labeled “Y,” the statistics derived or calculated are labeled “C,” and the unavailable or not required ones are labeled “-.”

## Supplementary Table 2. Statistics of Wood Consumption Fixed Effects Models

| **Model** | **Country Group** | **Country Number** | **$R^2$ Full** | **F-statistic Full** | **$R^2$ Proj** | **F-statistic Proj** | **RSE** | **Sample Size** | **DF** |
|---|---|---|---|---|---|---|---|---|---|
| ***log(SNW_WBP) ~ log(GDP_pcap) + log(POP) + NYEAR + NYEARS*** | GDP per cap > US$40,000 | 29 | 0.98 | 2088.8 | 0.33 | 146.6 | 0.32 | 1218 | 1185 |
| | GDP per cap < US$40,000 | 166 | 0.88 | 295.4 | 0.28 | 647.6 | 0.83 | 6811 | 6641 |
| ***log(PPB) ~ log(GDP_pcap) + log(POP) + NYEAR + NYEARS*** | GDP per cap > $12,000 | 67 | 0.98 | 1789.3 | 0.65 | 1175.4 | 0.39 | 2635 | 2564 |
| | GDP per cap < US$12,000 | 121 | 0.92 | 400.7 | 0.57 | 1412.8 | 0.82 | 4326 | 4201 |
| ***log(WFL) ~ log(GDP_pcap) + log(POP) + NYEAR + NYEARS*** | GDP per cap > US$12,000 | 64 | 0.95 | 736.7 | 0.10 | 76.5 | 0.65 | 2751 | 2683 |
| | GDP per cap < US$12,000 | 119 | 0.98 | 1801.8 | 0.19 | 316.2 | 0.40 | 5580 | 5457 |
| ***log(SNW_WBP) ~ log(GDP_pcap) + log(POP)*** | GDP per cap > US$40,000 | 29 | 0.98 | 2076.8 | 0.28 | 233.2 | 0.33 | 1218 | 1187 |
| | GDP per cap < US$40,000 | 166 | 0.88 | 293.0 | 0.27 | 1213.4 | 0.84 | 6811 | 6643 |
| ***log(PPB) ~ log(GDP_pcap) + log(POP)*** | GDP per cap > US$12,000 | 67 | 0.98 | 1754.2 | 0.63 | 2180.5 | 0.40 | 2635 | 2566 |
| | GDP per cap < US$12,000 | 121 | 0.92 | 403.4 | 0.57 | 2781.9 | 0.83 | 4326 | 4203 |
| ***log(WFL) ~ log(GDP_pcap) + log(POP)*** | GDP per cap > US$12,000 | 64 | 0.95 | 710.4 | 0.04 | 60.7 | 0.68 | 2751 | 2685 |
| | GDP per cap < US$12,000 | 119 | 0.98 | 1824.3 | 0.18 | 617.9 | 0.40 | 5580 | 5459 |

*Notes:* NYEAR = number of years from 1961; NYEAR = number of years from 2000 for shifting technology; GDP = gross domestic product; proj = projection; RSE = residual standard error; DF = degrees of freedom. The p values for all the models are close to zero (less than 2.2e-16).

## Supplementary Table 3. Fixed Effects Model Coefficients Statistics

| Model | Country Group | Variables | Coef. | Std. Error | t value | Pr(>\|t\|) | Lower limit (95% CI) | Upper limit (95% CI) |
|---|---|---|---|---|---|---|---|---|
| ***log(SNW_WBP) ~ log(GDP_pcap) + log(POP) + NYEAR + NYEARS*** | GDP per cap > US$40,000 | log(GDP_pcap) | 0.465 | 0.051 | 9.1 | 3.9E-19 | 0.365 | 0.566 |
| | | log(POP) | 0.968 | 0.066 | 14.7 | 4.2E-45 | 0.839 | 1.097 |
| | | NYEARS | -0.015 | 0.003 | -5.5 | 4.0E-08 | -0.020 | -0.009 |
| | | NYEAR | -0.004 | 0.002 | -2.4 | 1.5E-02 | -0.008 | -0.001 |
| | GDP per cap < US$40,000 | log(GDP_pcap) | 0.692 | 0.036 | 19.2 | 9.4E-80 | 0.621 | 0.763 |
| | | log(POP) | 0.732 | 0.079 | 9.2 | 4.3E-20 | 0.576 | 0.888 |
| | | NYEARS | 0.028 | 0.003 | 10.2 | 2.1E-24 | 0.023 | 0.033 |
| | | NYEAR | -0.004 | 0.002 | -1.8 | 7.0E-02 | -0.008 | 0.000 |
| ***log(PPB) ~ log(GDP_pcap) + log(POP) + NYEAR + NYEARS*** | GDP per cap > US$12,000 | log(GDP_pcap) | 0.781 | 0.035 | 22.5 | 4.6E-102 | 0.713 | 0.850 |
| | | log(POP) | 1.418 | 0.057 | 24.7 | 6.5E-121 | 1.305 | 1.530 |
| | | NYEARS | -0.022 | 0.002 | -11.0 | 1.1E-27 | -0.026 | -0.018 |
| | | NYEAR | 0.007 | 0.001 | 4.7 | 2.2E-06 | 0.004 | 0.009 |
| | GDP per cap < US$12,000 | log(GDP_pcap) | 1.168 | 0.052 | 22.5 | 4.5E-106 | 1.066 | 1.270 |
| | | log(POP) | 1.372 | 0.126 | 10.9 | 4.4E-27 | 1.124 | 1.620 |
| | | NYEARS | -0.014 | 0.003 | -4.1 | 3.5E-05 | -0.021 | -0.007 |
| | | NYEAR | 0.022 | 0.004 | 6.1 | 1.3E-09 | 0.015 | 0.029 |
| ***log(WFL) ~ log(GDP_pcap) + log(POP) + NYEAR + NYEARS*** | GDP per cap > US$12,000 | log(GDP_pcap) | -0.491 | 0.044 | -11.2 | 1.4E-28 | -0.577 | -0.405 |
| | | log(POP) | 0.111 | 0.069 | 1.6 | 1.1E-01 | -0.024 | 0.247 |
| | | NYEARS | 0.019 | 0.003 | 5.7 | 1.8E-08 | 0.012 | 0.025 |
| | | NYEAR | 0.012 | 0.002 | 6.1 | 1.6E-09 | 0.008 | 0.016 |
| | GDP per cap < US$12,000 | log(GDP_pcap) | -0.123 | 0.020 | -6.3 | 3.3E-10 | -0.162 | -0.085 |
| | | log(POP) | 0.491 | 0.045 | 10.9 | 3.7E-27 | 0.402 | 0.580 |
| | | NYEARS | 0.006 | 0.001 | 4.1 | 3.9E-05 | 0.003 | 0.009 |
| | | NYEAR | 0.001 | 0.001 | 0.4 | 6.9E-01 | -0.002 | 0.003 |
| ***log(SNW_WBP) ~ log(GDP_pcap) + log(POP)*** | GDP per cap > US$40,000 | log(GDP_pcap) | 0.279 | 0.030 | 9.2 | 2.3E-19 | 0.219 | 0.338 |
| | | log(POP) | 0.647 | 0.057 | 11.3 | 3.5E-28 | 0.535 | 0.759 |
| | GDP per cap < US$40,000 | log(GDP_pcap) | 0.805 | 0.030 | 26.9 | 1.2E-151 | 0.747 | 0.864 |
| | | log(POP) | 0.842 | 0.036 | 23.2 | 9.6E-115 | 0.771 | 0.913 |
| ***log(PPB) ~ log(GDP_pcap) + log(POP)*** | GDP per cap > US$12,000 | log(GDP_pcap) | 0.808 | 0.024 | 33.6 | 5.9E-205 | 0.761 | 0.856 |
| | | log(POP) | 1.321 | 0.051 | 26.1 | 2.7E-133 | 1.222 | 1.420 |
| | GDP per cap < US$12,000 | log(GDP_pcap) | 1.302 | 0.041 | 31.9 | 7.8E-200 | 1.222 | 1.382 |
| | | log(POP) | 2.010 | 0.051 | 39.2 | 2.9E-287 | 1.910 | 2.111 |
| ***log(WFL) ~ log(GDP_pcap) + log(POP)*** | GDP per cap > US$12,000 | log(GDP_pcap) | -0.178 | 0.033 | -5.4 | 9.3E-08 | -0.243 | -0.113 |
| | | log(POP) | 0.611 | 0.056 | 11.0 | 1.3E-27 | 0.503 | 0.720 |
| | GDP per cap < US$12,000 | log(GDP_pcap) | -0.085 | 0.016 | -5.3 | 1.6E-07 | -0.117 | -0.053 |
| | | log(POP) | 0.564 | 0.017 | 33.2 | 3.6E-220 | 0.531 | 0.597 |

*Notes:* Coef. = Coefficients; Std. = standard; Pr(>|t|) are p values; CI = Confidence Interval.

## Supplementary Table 4. Comparison of Global and Regional Timber Demand Projections

| | Long-Lived Products (LLP) | | Short-Lived Products (SLP) | Very-Short-Lived Products (VSLP) |
|---|---|---|---|---|
| | **Sawn wood** | **Wood-based panels** | **Paper and paperboard** | **Wood fuel** |
| **Kangas and Baudin 2003** | | **2000–20** | | |
| Europe | +24% (1.2%) | +38% (1.9%) | +50% (2.5%) | |
| **Szabó et al. 2009[8]** | | | **2000–30** | |
| Asia | | | +243% (8.1%) | |
| Europe | | | +44% (1.5%) | |
| North America | | | +36% (1.2%) | |
| South America | | | +200% (6.7%) | |
| **FAO 2009[9]** | | **2005–30** | | **2000–20** |
| Africa | +117% (4.7%) | +67% (2.7%) | +200% (8.0%) | +34% (1.7%) |
| East Asia and Pacific | +35% (1.4%) | +199% (7.9%) | +157% (6.3%) | –14% (–0.7%) |
| Europe | +41% (1.7%) | +74% (3.0%) | +78% (3.1%) | +536% (26.8%) |
| Latin America | +56% (2.3%) | +67% (2.7%) | +94% (3.8%) | +17% (0.9%) |
| North America | +34% (1.3%) | +64% (2.6%) | +56% (2.2%) | |
| Western and Central Asia | +77% (3.1%) | +211% (8.4%) | +150% (6.0%) | –30% (–1.5%) |
| World | +41% (1.6%) | +116% (4.6%) | +105% (4.2%) | |
| **Buongiorno 2015[10]** | | | **2015–50** | |
| East Asia and Pacific | +71% (2.0%) | | +62% (1.8%) | +9% (0.3%) |
| Europe and Central Asia | +22% (0.6%) | | +33% (0.9%) | +9% (0.3%) |
| Latin America | +40% (1.2%) | | +52% (1.5%) | +8% (0.2%) |
| Middle East and North Africa | +65% (1.9%) | | +49% (1.4%) | +9% (0.3%) |
| North America | +14% (0.4%) | | +29% (0.8%) | +9% (0.3%) |
| South Asia | +138% (3.9%) | | +137% (3.9%) | +5% (0.2%) |
| Sub-Saharan Africa | +48% (1.4%) | | +100% (2.9%) | –13% (–0.4%) |
| World | +46% (1.3%) | | +52% (1.5%) | +1% (0.0%) |
| **This study** | | | **2010–50** | |
| East Asia and Pacific | +177% (4.4%) | | +180% (5.6%) | +5% (0.1%) |
| Europe and Central Asia | +22% (0.5%) | | –7% (–0.2%) | –9% (–0.2%) |
| Latin America | +110% (2.7%) | | +249% (6.2%) | +8% (0.2%) |
| Middle East and North Africa | +169% (4.2%) | | +338% (8.5%) | +38% (0.9%) |
| North America | –28% (–0.7%) | | +3% (0.1%) | 5% (0.1%) |
| South Asia | +277% (6.9%) | | +904% (22.6%) | +18% (0.5%) |
| Sub-Saharan Africa | +317% (7.9%) | | +436% (10.9%) | +49% (1.2%) |
| World | +84% (2.1%) | | +128% (3.2%) | +22% (0.5%) |

*Note:* The linear annual growth rate (percentage per year) is in parentheses.

**Supplementary Table 5. Weighted Average National Forest Growth Parameters in the 30 Countries Used for the Global Analysis**

| Country | Young Secondary GR1 (tC $ha^{-1}$ $yr^{-1}$) | Middle-aged Secondary GR2 (tC $ha^{-1}$ $yr^{-1}$) | Average Secondary Carbon Stock (MgC/ha) | Existing Plantation GR (tC $ha^{-1}$ $yr^{-1}$) | FAO Plantation Area (ha) |
|---|---|---|---|---|---|
| **Australia** | 1.53 | 1.40 | 59.55 | 4.64 | 1,903,000 |
| **Austria** | 1.74 | 1.23 | 66.28 | 1.53 | 1,696,000 |
| **Bangladesh** | 3.43 | 1.14 | 88.61 | 2.74 | 237,000 |
| **Brazil** | 3.68 | 1.07 | 52.38 | 8.22 | 6,973,000 |
| **Canada** | 0.92 | 0.76 | 31.43 | 0.84 | 13,975,000 |
| **Chile** | 3.06 | 1.91 | 57.35 | 5.48 | 2,384,000 |
| **China** | 2.25 | 0.73 | 62.22 | 1.27 | 73,066,500 |
| **D.R. Congo** | 4.42 | 1.65 | 57.97 | 7.97 | 58,779 |
| **Ethiopia** | 2.75 | 0.79 | 61.97 | 5.82 | 511,000 |
| **Finland** | 0.89 | 0.61 | 27.77 | 0.86 | 6,775,401 |
| **France** | 1.83 | 1.30 | 79.99 | 1.73 | 2,086,000 |
| **Germany** | 1.68 | 1.26 | 81.32 | 1.73 | 5,290,000 |
| **Ghana** | 5.04 | 1.56 | 60.66 | 5.04 | 260,000 |
| **India** | 2.78 | 1.89 | 97.40 | 1.73 | 11,139,000 |
| **Indonesia** | 4.33 | 1.16 | 86.99 | 7.21 | 4,803,000 |
| **Japan** | 1.51 | 1.31 | 78.86 | 1.75 | 10,292,000 |
| **Kenya** | 3.37 | 0.75 | 54.79 | 3.37 | 193,000 |
| **Mexico** | 3.24 | 1.39 | 49.52 | 3.60 | 59,000 |
| **Myanmar** | 3.10 | 2.53 | 104.16 | 2.74 | 988,000 |
| **Nigeria** | 5.20 | 1.36 | 59.72 | 5.20 | 328,000 |
| **Pakistan** | 1.30 | 0.39 | 81.45 | 2.74 | 340,000 |
| **Poland** | 1.80 | 1.30 | 54.46 | 1.81 | 8,877,000 |
| **Russia** | 1.04 | 0.72 | 37.80 | 0.88 | 19,612,900 |
| **South Africa** | 1.74 | 0.81 | 59.97 | 3.59 | 1,763,000 |
| **Sweden** | 1.20 | 0.84 | 31.04 | 1.18 | 12,564,000 |
| **Thailand** | 3.96 | 2.04 | 93.75 | 3.70 | 3,986,000 |
| **Uganda** | 3.40 | 1.35 | 40.82 | 3.40 | 55,000 |
| **Tanzania** | 3.14 | 1.49 | 58.52 | 3.14 | 240,000 |
| **United States** | 2.11 | 1.09 | 61.46 | 3.05 | 25,564,000 |
| **Vietnam** | 3.38 | 2.62 | 82.34 | 6.74 | 3,823,000 |

*Note:* GR = growth rate. Average growth rates are based on a weighted average of growth rates of different secondary or plantation forests within each country.

## Supplementary Table 6. Summary of Sources Used for Plantation Rotation Periods

| Country | Rotation Period (years) | Description | Source |
|---|---|---|---|
| **Australia** | 20 | Applied short rotation length in line with other countries with similar growth rates | N/A |
| **Austria** | 100 | A long rotation of around 100 years | European Parliament[39] |
| **Bangladesh** | 30 | Applied same rotation length as neighboring countries with similar growth rates and more reliable data | N/A |
| **Brazil** | 7 | Rotation length range from 6-8 years (short rotation for Eucalyptus, Acacia) | Sanquetta et al.[40], le Maire et al.[41] |
| **Canada** | 100 | Chose a long rotation period due to the small growth rate, based on other countries at similar latitudes with similar growth rates | N/A |
| **Chile** | 20 | Based on the rotations of other countries with similar growth rates | N/A |
| **China** | 50 | National document provides rotation periods at a wide range of 16-80 years for normal wood usage; chose middle of this range | State Forestry Administration of China[42] |
| **D.R. Congo** | 7 | Applied short rotation length in tropical countries | N/A |
| **Ethiopia** | 7 | Applied short rotation length in tropical countries | N/A |
| **Finland** | 100 | National-level information suggests median rotation length of 100 years | NRIF[43] |
| **France** | 60 | Chose the same rotation length as neighboring countries with more reliable resources | N/A |
| **Germany** | 60 | Applied same rotation period as neighboring countries with similar growth rate and more reliable data | N/A |
| **Ghana** | 30 | Based on the rotations of other countries with similar growth rates | N/A |
| **India** | 50 | Applied average of 80 years for long rotation and 20 years for short | FAO[44] |
| **Indonesia** | 7 | Applied short rotation length in tropical countries | Harwood et al. [24] |
| **Japan** | 100 | Little information is available; rotation length was chosen based on small growth rate | N/A |
| **Kenya** | 30 | Based on the rotations of other countries with similar growth rates | N/A |
| **Mexico** | 30 | A country-level study suggests rotation lengths range from 50 to 80 years | Torres-Rojo et al.[45] |
| **Myanmar** | 20 | Applied a rotation length based on other countries with similar growth rates and more reliable data | N/A |
| **Nigeria** | 20 | Chose a rotation length based on the rotations of other countries with similar growth rates | N/A |
| **Pakistan** | 30 | Estimated rotation period based on relative proportion of long- to short-rotation forestry | UNDP[46] |
| **Poland** | 60 | Government document assumes 60-year rotation | Directorate General of the State Forests[47] |
| **Russia** | 100 | Chose a long rotation period in line with low growth rate | N/A |
| **South Africa** | 30 | A country-level study suggests rotation lengths range from 25-35 years | Crickmay & Associates[48] |
| **Sweden** | 80 | Country-level information suggests an average rotation of 80 years | InnoForest[49] |
| **Thailand** | 30 | based on the rotations of other countries with similar growth rates | N/A |
| **Uganda** | 20 | Applied a rotation period for other countries in sub-Saharan Africa with more reliable resources | N/A |
| **Tanzania** | 30 | Applied rotation length based on neighboring countries with more reliable data | N/A |
| **United States** | 32 | Based on weighted average at 32 years of likely rotation rates of 3 principal plantation types that make up 88% of estimated planted forest areas: 20 years for loblolly, 35 for longleaf, 80 years for Douglas fir | Hoover et al.[50] |
| **Vietnam** | 7 | Applied short rotation length in tropical countries | FAO[44], World Bank[51] |

*Notes:* N/A = not applicable.

**Supplementary Table 7. Secondary and Plantation Forest Slash Rates**

| Country | Secondary forest SR for LLP & SLP | Source | Plantation SR | Source |
|---|---|---|---|---|
| **Australia** | 30% | This study | 17% | BEF = 1.2 |
| **Austria** | 25% | This study | 13% | BEF = 1.15 |
| **Bangladesh** | 79% | Ellis et al.[30], Pearson et al. [31] | 33% | BEF = 1.5 |
| **Brazil** | 65% | Ellis et al.[30], Pearson et al. [31] | 13% | BEF = 1.15[a] |
| **Canada** | 25% | This study | 25% | Use natural slash rate at high efficiency |
| **Chile** | 79% | Ellis et al.[30], Pearson et al. [31] | 22% | BEF 1.2 for Pine and 1.5 for Eucalyptus[b] |
| **China** | 30% | This study | 19% | BEF = 1.15-1.5[b] |
| **D.R. Congo** | 82% | Ellis et al.[30], Pearson et al. [31] | 33% | BEF = 1.5 |
| **Ethiopia** | 64% | Ellis et al.[30], Pearson et al. [31] | 33% | BEF = 1.5 |
| **Finland** | 25% | This study | 25% | Use natural slash rate at high efficiency |
| **France** | 25% | This study | 13% | BEF = 1.15 |
| **Germany** | 25% | This study | 25% | Use natural slash rate at high efficiency |
| **Ghana** | 64% | Ellis et al.[30], Pearson et al. [31] | 25% | Use natural slash rate at high efficiency |
| **India** | 79% | Ellis et al.[30], Pearson et al. [31] | 33% | BEF = 1.5 |
| **Indonesia** | 79% | Ellis et al.[30], Pearson et al. [31] | 29% | BEF 1.33 for Acacia and 1.5 for Eucalyptus[b] |
| **Japan** | 30% | This study | 13% | BEF = 1.15 |
| **Kenya** | 64% | Ellis et al.[30], Pearson et al. [31] | 25% | Use natural slash rate at high efficiency |
| **Mexico** | 71% | Ellis et al.[30], Pearson et al. [31] | 24% | BEF = 1.05-1.5[b] |
| **Myanmar** | 79% | Ellis et al.[30], Pearson et al. [31] | 33% | BEF = 1.5 |
| **Nigeria** | 64% | Ellis et al.[30], Pearson et al. [31] | 25% | Use natural slash rate at high efficiency |
| **Pakistan** | 30% | This study | 33% | BEF = 1.5 |
| **Poland** | 25% | This study | 25% | Use natural slash rate at high efficiency |
| **Russia** | 30% | This study | 25% | Use natural slash rate at high efficiency |
| **South Africa** | 30% | This study | 25% | Use natural slash rate at high efficiency |
| **Sweden** | 25% | This study | 25% | Use natural slash rate at high efficiency |
| **Thailand** | 79% | Ellis et al.[30], Pearson et al. [31] | 33% | BEF = 1.5 |
| **Uganda** | 64% | Ellis et al.[30], Pearson et al. [31] | 25% | Use natural slash rate at high efficiency |
| **Tanzania** | 64% | Ellis et al.[30], Pearson et al. [31] | 25% | Use natural slash rate at high efficiency |
| **United States** | 25% | This study | 10% | BEF=1.1-1.15[b] |
| **Vietnam** | 79% | Ellis et al.[30], Pearson et al. [31] | 33% | BEF = 1.5 |

*Note:* SR = slash rate. a. see our discussion on Brazil plantation growth rate; b. the slash rate is a weighted average of main species based on area share

**Supplementary Table 8. Description of Carbon Pools in CHARM**

| Pool | Description | Half-Life (years) |
|---|---|---|
| **Stand** | Live aboveground and belowground biomass in the forest | N/A |
| **Slash** | Dead biomass that is left and decays following a harvest | 18[52] |
| **Dead roots** | Decaying roots from trees that have been harvested | 5.2[53,54] |
| **VSLP** | Very-short-lived products (biomass burned for energy immediately) | N/A |
| **SLP** | Short-lived products (paper products) | 2.5[55] |
| **LLP** | Long-lived products (timber used for furniture or construction); LLPs are subdivided into wood used for furniture and wood used for construction because of their different storage lives | 13–47[55] |
| **Landfill** | Temporary storage of LLPs that are disposed at the end of life | 29[56] |
| **Fossil carbon** | Changes in fossil carbon due to the use of wood as a substitute for alternative products | |

*Note:* N/A = not applicable.

**Supplementary Table 9. LLP Percentage in Construction**

| Country | % LLP used in construction |
|---|---|
| United States | 45 |
| Japan | 67 |
| United Kingdom | 14 |
| France | 32 |
| Germany | 30 |
| China | 59 |
| Russia | 17 |
| Finland | 56 |
| Sweden | 50 |
| Canada | 51 |
| All other LLP-producing countries | 42 |

*Note:* LLP = long-lived product.

**Supplementary Table 10. Half-Lives for LLPs in Construction and Other Uses**

| Country/Region | Half-Life for LLPs in Construction (years) | Half-Life for Other LLPs (years) |
|---|---|---|
| Canada | 66 | 29 |
| United States | 65 | 30 |
| Germany | 35 | 17 |
| Ireland | 67 | 30 |
| Finland | 21 | 23 (default) |
| France | 17 | 11 |
| Czech Republic | 45 | 23 (default) |
| Portugal | 21 | 14 |
| Switzerland | 55 | 35 |
| Spain | 17 | 12 |
| European Union (rest) | 43 | 27 |
| Japan | 33 | 20 |
| All other countries | 40 | 23 |

*Note:* LLP = long-lived product.

**Supplementary Table 11. Growth Rates in Harvest versus Non-harvest Scenarios**

| Scenario | | Initial Condition | Growth Function |
|---|---|---|---|
| ***(1) Allowing a secondary forest regrowth after harvest*** | Harvest scenario | Monod function at the age of harvest | Harvested once and grows at a Monod function |
| | Non-harvest scenario | Monod function at the age of harvest | Continue growing at Monod function |
| ***(2) Converting a secondary forest into a plantation*** | Harvest scenario | Monod function at the age of harvest | Harvested after each rotation cycle and grows at plantation growth rate |
| | Non-harvest scenario | Monod function at the age of harvest | Continue growing at Monod function |
| ***(3) Harvesting an existing plantation*** | Harvest scenario | Plantation carbon stock after one rotation cycle | Harvested after each rotation cycle and grows at plantation growth rate |
| | Non-harvest scenario | Monod function at the age of one plantation rotation cycle | Continue growing at Monod function |
| ***(4) Converting agricultural land into a plantation*** | Harvest scenario | Zero carbon stock | Harvested after each rotation cycle and grows at plantation growth rate |
| | Non-harvest scenario | Zero carbon stock | Grows at Monod function |

**Supplementary Table 12. Sensitivity Analysis of Secondary Forest Growth Rates**

| Experiments | Description | BAU gross emissions ($GtCO_2e$) | Carbon % Change | BAU land requirements (Mha) | Land % Change |
|---|---|---|---|---|---|
| Baseline | Default simulation | 4.11 | | 855.13 | |
| Both GRs 25% Up | Increase both growth rates by 25% | 4.64 | 13.0% | 766.97 | -10.3% |
| Both GRs 25% Down | Decrease both growth rates by 25% | 3.60 | -12.4% | 960.59 | 12.3% |
| GR1/GR2 25% Up | Increase the ratio of young forest growth rate to older forest growth rate by 25% | 3.69 | -10.2% | 851.82 | -0.4% |
| GR1/GR2 25% Down | Reduce the ratio of young forest growth rate to older forest growth rate by 25% | 4.64 | 13.1% | 849.02 | -0.7% |
| GR1/GR2 50% Up | Increase the ratio of young forest growth rate to older forest growth rate by 50% | 3.36 | -18.1% | 849.97 | -0.6% |

**Supplementary Table 13. Sensitivity Analysis of Root to Shoot Ratio**

| Experiments | Description | BAU gross emissions ($GtCO_2e$) | Carbon % Change | BAU land requirements (Mha) | Land % Change |
|---|---|---|---|---|---|
| Baseline | Default simulation | 4.11 | | 855.13 | |
| Root/Shoot 25% Up | Increase root to shoot ratio by 25% | 4.33 | 5.6% | 855.13 | 0.0% |
| Root/Shoot 25% Down | Decrease root to shoot ratio by 25% | 3.88 | -5.6% | 855.13 | 0.0% |

## Supplementary Table 14. Sensitivity Analysis of Choice of Economic Growth Model

| Experiments | Additional BAU wood production increase (dry matter million tons) | % Change | BAU emissions ($GtCO_2e$) | Additional BAU emissions ($GtCO_2e$) | Carbon % Change | BAU land (Mha) | Additional BAU land area (Mha) | Land % Change |
|---|---|---|---|---|---|---|---|---|
| Baseline | 20,670 | | 4.11 | 0.89 | | 855.13 | 218.65 | |
| OECD | 24,461 | 18% | 4.23 | 1.01 | 13.5% | 909.04 | 272.57 | 24.7% |
| IIASA | 24,831 | 20% | 4.29 | 1.07 | 20.3% | 915.54 | 279.06 | 27.6% |
| LINE | 17,397 | -16% | 4.00 | 0.78 | -12.0% | 806.91 | 170.44 | -22.1% |

## Supplementary Table 15. Sensitivity Analysis of Altering Trade Patterns

| Experiments | Description | BAU gross emissions ($GtCO_2e$) | Carbon % Change | BAU land requirements (Mha) | Land % Change |
|---|---|---|---|---|---|
| Baseline | Default simulation | 4.11 | | 855.13 | |
| Tropical exports 50% Up | Increase top tropical countries' export share by 50% | 4.10 | -0.2% | 853.07 | -0.2% |
| Tropical exports 50% Down | Decrease top tropical countries' export share by 50% | 4.11 | 0.2% | 857.27 | 0.3% |

## C. List of Abbreviations

| | |
|---|---|
| AGB | aboveground biomass |
| BAU | business as usual |
| BEF | biomass expansion factor |
| Bha | billon hectares |
| BGB | belowground biomass |
| C&S | concrete and steel |
| CHARM | Carbon Harvest Model |
| CLT | cross-laminated timber |
| $CO_2e$ | carbon dioxide equivalent |
| CW | carbon fraction of dry wood |
| DM | dry matter |
| FAO | Food and Agriculture Organization of the United Nations |
| FE | fixed effects |
| GHG | greenhouse gas |
| GR1 | growth rate of less than 20 years of age |
| GR2 | growth rate of greater than 20 years of age |
| GtC | gigaton of carbon |
| $GtCO_2e$ | gigaton of carbon dioxide equivalent |
| GWP | global warming potential |
| IIASA | International Institute for Applied Systems Analysis |
| IND | industrial roundwood |
| IND-M | main industrial roundwood |
| IND-O | other industrial roundwood |
| IND-PS | industrial roundwood used for pulping and sawing |
| IPCC | Intergovernmental Panel on Climate Change |
| LINE | linear extrapolation |
| LLP | long-lived product |
| LLP-M | main long-lived product |
| LLP-O | other long-lived product |
| LPG | liquefied petroleum gas |
| MAI | mean annual increment |
| MgC | megagram of carbon |
| Mha | million hectares |
| OECD | Organisation for Economic Co-operation and Development |
| OSB | oriented strand board |
| PDV | present discount value |
| PPB | paper and paperboard |
| PS | pulp and sawn |
| RSE | residual standard error |
| SF | substitution factor |
| SLP | short-lived product |
| SNW | sawn wood |
| SR | slash rate |
| SSP | Shared Socioeconomic Pathway |

| | |
|---|---|
| tC | tons of carbon |
| UNEP | United Nations Environment Programme |
| VSLP-IND | very-short-lived product, industrial waste |
| VSLP-IND-O | very-short-lived product, other industrial roundwood waste |
| VSLP-WFL | very-short-lived product, wood fuel |
| WBD | wood basic density |
| WBP | wood-based panels |
| WFL | wood fuel |
| WPL | wood pulp |

## D. Supporting Information References


1. FAO. *FAOSTAT Forestry Production and Trade domain* http://www.fao.org/faostat/en/#data/FO (FAO, 2021).
2. FAO, ITTO and U. N. *Forest product conversion factors*. doi:10.4060/ca7952en (FAO, ITTO and United Nations, 2020)..
3. Wooldridge, J. M. *Econometric analysis of cross section and panel data*. (MIT press, 2010).
4. Dellink, R., Chateau, J., Lanzi, E. & Magné, B. Long-term economic growth projections in the Shared Socioeconomic Pathways. *Glob. Environ. Change* **42**, 200–214 (2017).
5. Cuaresma, J. C. Income projections for climate change research: A framework based on human capital dynamics. *Glob. Environ. Change* **42**, 226–236 (2017).
6. U.S. Inflation Calculator.
7. UNDESA. World Population Prospects: The 2019 Revision. https://population.un.org/wpp/ (2019).
8. Szabó, L., Soria, A., Forsström, J., Keränen, J. T. & Hytönen, E. A world model of the pulp and paper industry: Demand, energy consumption and emission scenarios to 2030. *Environ. Sci. Policy* **12**, 257–269 (2009).
9. Perlis, A. State of the world's forests 2009. *Rome Food Agric. Organ. FAO* (2009).
10. Buongiorno, J. Global modelling to predict timber production and prices: the GFPM approach. *For. Int. J. For. Res.* **88**, 291–303 (2015).
11. Hurmekoski, E. & Hetemäki, L. Studying the future of the forest sector: Review and implications for long-term outlook studies. *For. Policy Econ.* **34**, 17–29 (2013).
12. Chiba, T., Oka, H. & Kayo, C. Socioeconomic factors influencing global paper and paperboard demand. *J. Wood Sci.* **63**, 539–547 (2017).
13. Harris, N. L. *et al.* Global maps of twenty-first century forest carbon fluxes. *Nat. Clim. Change* **11**, 234–240 (2021).

14. Bernal, B., Murray, L. T. & Pearson, T. R. H. Global carbon dioxide removal rates from forest landscape restoration activities. *Carbon Balance Manag.* **13**, 22 (2018).
15. McMahon, S. M., Parker, G. G. & Miller, D. R. Evidence for a recent increase in forest growth. *Proc. Natl. Acad. Sci.* **107**, 3611–3615 (2010).
16. Poorter, L. *et al.* Biomass resilience of Neotropical secondary forests. *Nature* **530**, 211–214 (2016).
17. Harris, N., Goldman, E. D. & Gibbes, S. Spatial database of planted trees (SDPT) version 1.0. *Tech. Note World Resour. Inst.* (2019).
18. IPCC. *Good practice guidance for land use, land-use change and forestry*. (Institute for Global Environmental Strategies (IGES), 2003).
19. IBA. *Brazilian Tree Industry*. (2014).
20. IBA. *Brazilian Tree Industry*. (2022).
21. Liu, Y., Gao, X. L., Fu, C., Yu, G. R. & Liu, Z. Y. Estimation of carbon sequestration potential of forest biomass in China based on National Forest Resources Inventory. *Acta Ecol. Sin.* **39**, 4002–401 (2019).
22. State Forestry and Grassland Administration. Xi Jinping Delivers a Video Speech at the Opening Ceremony of the BRICS Foreign Ministers' Meeting. (2022).
23. Zeng, W. Developing One-Variable Individual Tree Biomass Models Based on Wood Density for 34 Tree Species in China. *For. Resour. Manag.* **0**, 41–46 (2017).
24. Harwood, C. E. & Nambiar, E. K. S. Productivity of acacia and eucalypt plantations in Southeast Asia. 2. trends and variations. *Int. For. Rev.* **16**, 249–260 (2014).
25. Miyakuni, K., Heriansyah, I., Heriyanto, N. M. & Kiyono, Y. Allometric Biomass Equations, Biomass Expansion Factors and Root-to-shoot Ratios of Planted *Acacia mangium* Willd. Forests in West Java, Indonesia. *J. For. Plan.* **10**, 69–76 (2004).
26. Prasetyo, A. *et al.* Variations on growth characteristics and wood properties of three *Eucalyptus* species planted for pulpwood in Indonesia. *Tropics* **26**, 59–69 (2017).

27. Fierros, M. Programa de Desarrollo de Plantaciones Forestales Comerciales: a 15 años de su creación. *CONAFOR Jalisco México* (2012).

28. Oswalt, S. N., Smith, W. B., Miles, P. D. & Pugh, S. A. Forest Resources of the United States, 2017: a technical document supporting the Forest Service 2020 RPA Assessment. *Gen Tech Rep WO-97 Wash. DC US Dep. Agric. For. Serv. Wash. Off.* 97 (2019).

29. Hoover, C. M., Bagdon, B. & Gagnon, A. *Standard estimates of forest ecosystem carbon for forest types of the United States*. 1–158 https://www.fs.usda.gov/research/treesearch/63638 (2021).

30. Ellis, P. W. *et al.* Reduced-impact logging for climate change mitigation (RIL-C) can halve selective logging emissions from tropical forests. *For. Ecol. Manag.* **438**, 255–266 (2019).

31. Pearson, T. R. H., Brown, S., Murray, L. & Sidman, G. Greenhouse gas emissions from tropical forest degradation: an underestimated source. *Carbon Balance Manag.* **12**, 3 (2017).

32. Eggleston, H., Buendia, L., Miwa, K., Ngara, T. & Tanabe, K. 2006 IPCC guidelines for national greenhouse gas inventories. (2006).

33. Zhang, X., Chen, J., Dias, A. C. & Yang, H. Improving carbon stock estimates for in-use harvested wood products by linking production and consumption—a global case study. *Environ. Sci. Technol.* **54**, 2565–2574 (2020).

34. Smyth, C., Rampley, G., Lemprière, T. C., Schwab, O. & Kurz, W. A. Estimating product and energy substitution benefits in national-scale mitigation analyses for Canada. *Gcb Bioenergy* **9**, 1071–1084 (2017).

35. Chen, J., Ter-Mikaelian, M. T., Yang, H. & Colombo, S. J. Assessing the greenhouse gas effects of harvested wood products manufactured from managed forests in Canada. *For. Int. J. For. Res.* **91**, 193–205 (2018).

36. Pendrill, F. *et al.* Agricultural and forestry trade drives large share of tropical deforestation emissions. *Glob. Environ. Change* **56**, 1–10 (2019).

37. Gough, C. M., Curtis, P. S., Hardiman, B. S., Scheuermann, C. M. & Bond-Lamberty, B. Disturbance, complexity, and succession of net ecosystem production in North America's temperate deciduous forests. *Ecosphere* **7**, e01375 (2016).
38. Mokany, K., Raison, R. J. & Prokushkin, A. S. Critical analysis of root : shoot ratios in terrestrial biomes. *Glob. Change Biol.* **12**, 84–96 (2006).
39. European Parliament. Austria: Forest Structure. in *Europe and the Forest.* vol. Vol. 3 (1997).
40. Sanquetta, C. R. *et al.* Dynamics of carbon and CO2 removals by Brazilian forest plantations during 1990–2016. *Carbon Balance Manag.* **13**, 20 (2018).
41. le Maire, G., Dupuy, S., Nouvellon, Y., Loos, R. A. & Hakamada, R. Mapping short-rotation plantations at regional scale using MODIS time series: Case of eucalypt plantations in Brazil. *Remote Sens. Environ.* **152**, 136–149 (2014).
42. State Forestry Administration of China. Regulations for age-class and age-group division of main tree-species. (2018).
43. Natural Resources Institute Finland. Criterion 1: Forest Resources. (2012).
44. FSIV (Forest Science Institute of Vietnam). *Vietnam Forestry Outlook Study*. https://www.fao.org/3/am254e/am254e00.pdf (2009).
45. Torres-Rojo, J. M., Moreno-Sánchez, R. & Mendoza-Briseño, M. A. Sustainable forest management in Mexico. *Curr. For. Rep.* **2**, 93–105 (2016).
46. UNDP (United Nations Development Programme. *Forests & Biodiversity Information/Data Report.* (2013).
47. Directorate General of the State Forests. *Forests in Poland 2017.* http://www.lasy.gov.pl/pl/informacje/publikacje/in-english/forests-in-poland/lasy-w-polsce-2017-en.pdf. (2017).
48. Crickmay & Associates. Supply and demand study of softwood sawlog and sawn timber in South Africa. (2004).

49. Hertog, I., Brogaard, S. & Krause, T. The Swedish forestry model: intensifying production for sustainability. (2019).

50. Hoover, C. M. & Smith, J. E. Current aboveground live tree carbon stocks and annual net change in forests of conterminous United States. *Carbon Balance Manag.* **16**, 574 (2021).

51. World Bank. *Forest Country Note: Vietnam https://documents1.worldbank.org/curated/en/544891570456214844/pdf/Vietnam-Country-Forest-Note.pdf.* (2019).

52. Russell, M. B. *et al.* Residence Times and Decay Rates of Downed Woody Debris Biomass/Carbon in Eastern US Forests. *Ecosystems* **17**, 765–777 (2014).

53. Brunner, I. & Godbold, D. L. Tree roots in a changing world. *J. For. Res.* **12**, 78–82 (2007).

54. Zhang, X. & Wang, W. The decomposition of fine and coarse roots: their global patterns and controlling factors. *Sci. Rep.* **5**, 9940 (2015).

55. Pingoud, K. & Wagner, F. Methane Emissions from Landfills and Carbon Dynamics of Harvested Wood Products: The First-Order Decay Revisited. *Mitig. Adapt. Strateg. Glob. Change* **11**, 961–978 (2006).

56. Skog, K. E. Sequestration of carbon in harvested wood products for the United States. *For. Prod. J.* **58**, 56-72 (2008).
